# Interventions against loneliness and social isolation in older adults– a systematic review

**DOI:** 10.1186/s12889-026-27683-9

**Published:** 2026-05-18

**Authors:** Johanna Bergsträsser, Teresa Schmahl, Jost Steinhäuser, Katja Goetz

**Affiliations:** Institute of Family Medicine, University of Luebeck , Luebeck, Germany

**Keywords:** Loneliness, Social isolation, Older adults, Ageing, Intervention, COVID-19, Systematic review

## Abstract

**Background:**

Loneliness is a growing health and social challenge. The COVID-19 pandemic boosted feelings of loneliness for many people. To combat loneliness, a wide range of interventions have been developed and evaluated for their efficacy and effectiveness. As a result, many new interventions to alleviate loneliness have been developed, especially technological interventions. Therefore, the objective of this study was to conduct a comprehensive and up—to-date systematic review to identify interventions aimed at loneliness and social isolation among adults, 60 years and older published during or post-pandemic.

**Methods:**

A comprehensive literature search was conducted for studies between 2020 and 2024 across five online databases: MEDLINE via PubMed, Web of Science, Scopus, Cochrane Library, and CINAHL. Title and abstract screening, critical appraisal of the studies, and data extraction were performed by two independent reviewers. A narrative approach was adopted to assess and integrate the diverse findings from the research.

**Results:**

Ultimately, 79 studies were included in this systematic review. The results are structured based on the categorization of the interventions, which includes differentiation between analogue interventions, technological interventions and multicomponent (analogue and technological) interventions. The effectiveness of analogue interventions, particularly community-based interventions such as group meetings, social participation programs, and educational or psychological interventions, tended to be superior to that of technological interventions.

**Conclusions:**

The combination of analogue and technological interventions in particular produced promising results regarding a decrease of loneliness. Specific interventions must be tailored to the target group and setting and regularly reevaluated. The aim now should be to implement these interventions comprehensively and monitor their effectiveness over several years. Future research should focus on differentiating the circumstances under which various forms of intervention are effective.

**Trial registration:**

PROSPERO systematic review registration: CRD42024538755.

**Supplementary Information:**

The online version contains supplementary material available at 10.1186/s12889-026-27683-9.

## Background

Loneliness represents increasing health and social challenges, particularly among older adults [1–3]. In China, Europe, and the United States, 20—34% of older adults are identified as experiencing loneliness [3]. Loneliness is described as a negative and stressful emotional state that arises from the discrepancy between a person’s desired and actual social relationships [4, 5]. Loneliness is more closely associated with the quality than the number of relationships [6]. Distinguishing itself from the subjective state of loneliness, social isolation can be defined as an “objective lack of social contact with others” [2]. Both concepts are considered into the research due to their similarities and partially synonymous use in the literature.

High levels of loneliness have been found among people with mental illness, hearing and visual impairments, and chronic health problems observed [7, 8]. Loneliness often occurs during the transition process from middle to old age, when social roles are broken down and various changes occur at an individual and family level [2, 9]. This phase is usually characterized by the end of work life and the transition to retirement. At the same time, this phase is emotionally marked by the loss of family members and friendships [10]. Additionally, factors such as lack of a partner, low levels of social activity, and poor self-perceived health are identified as risk factors associated with loneliness [7, 11]. Loneliness can have a negative impact on the quality of life of those affected and they are associated with increased morbidity and predispose individuals to cardiovascular diseases, strokes, diabetes, dementia or depression [2, 12–14]. Moreover, an increased mortality rate has been observed among lonely and socially isolated people [15–17]. Due to these health and life-threatening risks, managing loneliness must be construed as a societal imperative. This includes raising public awareness, augmenting research activities on loneliness prevention and alleviation and providing accessible interventions for those affected. Consequently, a wide range of interventions against loneliness and social isolation have been developed and evaluated for their efficacy [18–22]. They underscored the importance of tailoring potential interventions to the needs of the individuals and social groups affected [19].

Numerous studies have been published focusing on interventions targeting specifically social isolation [18, 20, 21]. The findings suggest that group interventions and person-centered interventions over longer periods have particularly beneficial effects on reducing social isolation [20].

Reviews on this topic have been published previously, reporting varying results regarding different forms of intervention. The efficacy of group interventions has been highlighted in several reviews [18, 20]. Additionally, the potential of technological interventions to combat loneliness has been emphasized [21–23]. However, the quality of the evidence base has been rated as insufficient in several reviews, underscoring the need for further research [18, 21, 22, 24].

The COVID-19 pandemic has had a multifaceted impact on the proliferation, perception, and management of loneliness [22]. The pandemic has caused or exacerbated loneliness and social isolation in many people [25, 26]. In particular, the restrictions imposed by the COVID-19 pandemic (staying at home) have increased loneliness and social isolation [27, 28]. The heightened demand during the pandemic has spurred further development and improvement of digital offerings and internet-based interventions. Consequently, results from several studies suggest that technological interventions may be associated with lower levels of perceived loneliness [22]. To evaluate the longer-term impact of the pandemic on effective interventions against loneliness, it is imperative to update existing results. Therefore, the objective of this study was to conduct a comprehensive and up—to-date systematic literature review to identify interventions aimed at loneliness and social isolation among adults, 60 years and older, regardless of specific diseases and comorbidities published during or post-pandemic.

## Methods

The report of this Systematic Review adhered to the “Preferred Reporting Items for Systematic Reviews and Meta-analyses” (PRISMA) guidelines and utilized the corresponding checklist [29] (Additional file 1 Table 1: PRISMA Checklist). Additionally, in accordance with the study protocol, this systematic review was prospectively registered with PROSPERO (CRD42024538755). An initial search of the Cochrane Database of systematic reviews, MEDLINE (PubMed) and Google Scholar did not reveal any current or ongoing scoping reviews or systematic reviews on this topic.

The process of creating the review was undertaken by the authors JB (Johanna Bergsträsser, medical student and PhD student) and TS (researcher and health scientist) to ensure the validity of the research.

### Eligibility criteria

At the beginning, the inclusion and exclusion criteria were established based on the research question and the PICO scheme (Population, Intervention, Control, Outcome) (Additional file 2 Table 2: Eligibility criteria). Given the heightened prevalence of loneliness in older adults, a minimum age of 60 years was established to explore tailored interventions for this specific population. Recognizing social isolation and loneliness as distinct concepts often used interchangeably, both terms were integrated into the search. The focus was on interventions targeting loneliness and promoting social integration to mitigate perceived loneliness or social isolation. Consequently, retrospective and prospective longitudinal studies, randomised control trials, cross-sectional studies, observational studies, qualitative studies and case studies were included.

Studies focusing on specific populations or addressing specific diseases or comorbidities to ensure the widest possible applicability of the results in society were excluded. Interventions which focused on physically healthy older adults were included into the search. Additionally, settings such as nursing homes, care homes, and assisted living facilities were excluded. Non-original studies such as poster presentations, opinions, book reviews, expert reports, letters, comments, editorials, abstracts, protocols, trail registration and case studies were also excluded. Reviews meeting the inclusion criteria underwent screening for original research articles, which were then included instead of the reviews. As the aim of this study is to identify the situation after the pandemic and the development of interventions against loneliness in recent years, the search was limited to studies published between 2020 and 2024. Furthermore, we focused on studies available in English or German language.

### Search strategy

The search strategy was collaboratively developed and adapted to all databases by both authors (JB, TS). Initially, search terms were generated through brainstorming sessions, keyword searches, and analysis of MeSH terms in databases. Synonyms for the search terms and keywords from relevant articles were also incorporated. Various search terms were evaluated for relevance, refined, or eliminated from the search. This iterative process continued until the final version of the search strategy was established (Additional file 3 Table 3: Search strategy).

### Information sources

A comprehensive multi-database literature search was conducted in March and June 2024. The last day of the search was June 14th, 2024. The following databases were included on the search: Medline, Scopus, CINAHL, Web of Science Core Collection, and Cochrane Library. Additionally, relevant publications in several journals and Google Scholar were manually searched to identify studies that may not have been registered in the selected databases. Furthermore, the reference lists of the articles included in this review were examined to identify any additional eligible publications. Forward and backward citation chasing was also performed on studies identified as included at full text, ensuring comprehensive retrieval of relevant studies.

### Selection process

The title, abstract and full text screening followed predefined inclusion and exclusion criteria. Two independent researchers (JB, TS) conducted the process of study selection. At each step, any discrepancies were discussed, and consensus was reached. In cases of persisting conflicts, a third researcher (KG or JS) intervened as an independent supervisor to resolve them. The screening process was carried out using Rayyan [30].

### Data collection process and data items

A data extraction table was used for the extraction of appropriate studies. Data extraction was conducted by two researcher (JB, TS) and recorded in a standardized Excel template. The data extraction process encompassed multiple elements, including title, authors, year of publication, study aim, study design, study population, intervention, control, outcome parameters, measurement, time points, main results, and key conclusion. The studies were categorized based on the interventions conducted. The specific elements were adapted based on the findings and characteristics of the studies retrieved.

### Study risk of bias assessment

The authors (JB, TS) used different tools to assess the risk of bias depending on the study design. This approach included the use of RoB 2 tool for randomized trials [31], the ROBINS-I tool for nonrandomized studies [32], the Checklist for Qualitative Research of the Joanne Briggs Institute for qualitative studies [33] and the Mixed Methods Appraisal Tool [31]. No studies were excluded based on their risk scores. Any discrepancies in the assessment of bias were resolved through consensus between the two reviewers or by consulting a third reviewer (KG).

### Synthesis methods

Data analysis was conducted by both researchers (JB, TS) between March and June 2024. This review examined both qualitative and quantitative evidence concerning interventions for mitigating loneliness and social isolation. Meta-analytic techniques were deemed inappropriate due to the heterogeneity observed in the interventions studied and the extracted outcome data. Consequently, a narrative approach was adopted to assess and integrate the diverse findings from the research.

## Results

### Study selection

**Fig. 1:**
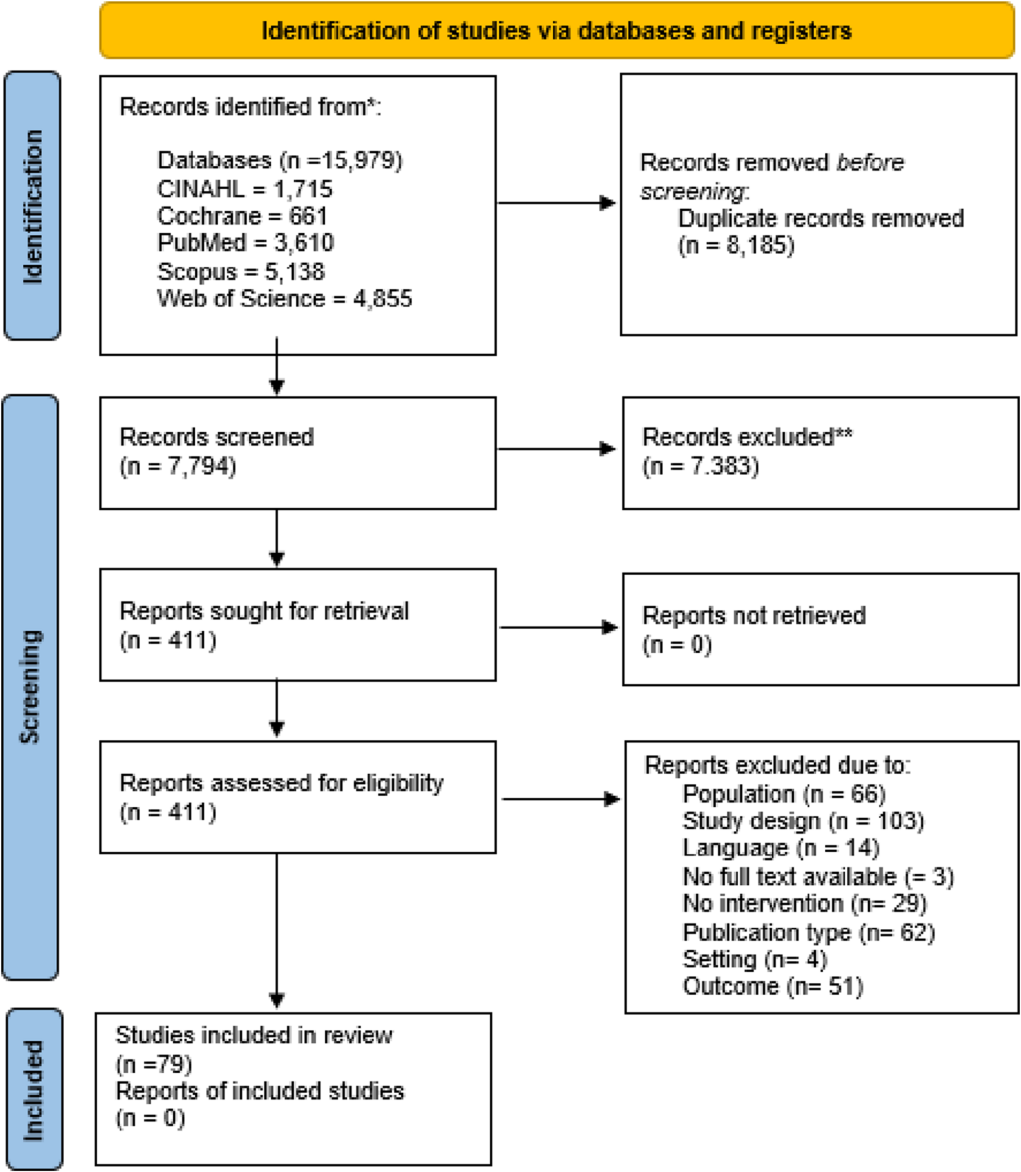
PRISMA flow diagram

The search strategy identified a total of 15,979 studies in all databases, with 8,185 duplicates removed. Title and abstract screening of 7,794 studies was performed, excluding 7.383 studies. Full-text screening was subsequently conducted for the remaining 411 studies, resulting in an exclusion of 332 additional studies. Ultimately, 79 studies met the inclusion criteria for the systematic review. The primary reasons for exclusion were inappropriate study designs (e.g., reviews or study protocols) and unsuitable study populations (e.g., age, setting or comorbidities). The corresponding PRISMA flow diagram is presented in Fig. 1.

### Study characteristics

The included studies were published between 2020 and 2024, with sample sizes ranging from 4 to 1012 participants and age from 60 to 99 years. Most studies included participants of all genders (74 studies), with a predominance of female participants. Numerous settings were represented, including living in communities, living alone, and daycare centers. The majority of studies were conducted in the United States (20 studies) and Canada (7 studies), followed by Asian countries such as South Korea (5 studies), Taiwan (4 studies) and India (2 studies). The review included 24 randomized controlled trials (RCTs), 39 non-randomized studies, six qualitative studies and 10 studies utilizing a mixed-methods design.

Loneliness was predominantly measured using the UCLA loneliness scale (55 studies), with most studies employing the 20-item version (32 studies).

### Measurement of loneliness

Loneliness and other outcome parameters, such as social isolation and social participation, were measured using a variety of assessments. A summary of all assessments is provided (Additional file 4 Table 4: Outcome parameters).

### Risk of bias in studies

**Fig. 2:**
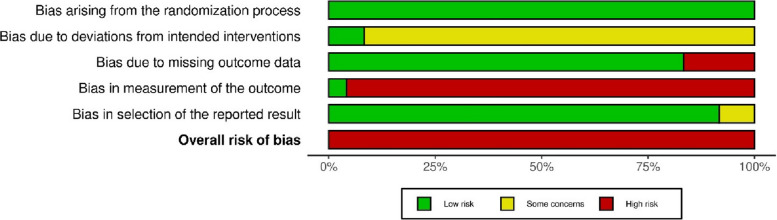
Summary plot—reporting the results of the RoB2 tool

Among the 24 included randomized controlled studies, all were assessed to be at high risk of bias according to the RoB 2 tool (Fig. 2, Additional file 5 Fig.1: RoB2 Traffic light plot). This is mainly due to the fact that the domain "bias in measurement of the outcome" was rated as high risk because the outcome measurement was self-administered and therefore not blinded. The frequent rating of "some concerns" in the area of "bias due to deviations from intended interventions" was also striking, as both the participants and the carers and people delivering the interventions were not blinded and the patients therefore knew which group they were assigned.

**Fig. 3:**
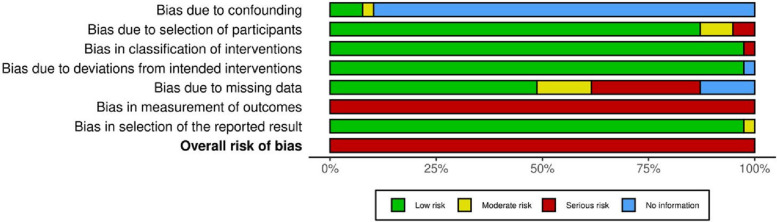
Summary plot—reporting the results of the ROBINS-I tool

The non-randomized studies were uniformly assessed as having a serious risk of bias due to similar reasons (Fig. 3, Additional file 6 Fig. 2: ROBINS-I Traffic light plot). Furthermore, potential confounders were only mentioned in only a few studies, resulting in an assessment of “no information” in this domain.

The overall quality of the mixed methods studies is between sufficient and good, with the qualitative category achieving very good ratings (Table 1). Most of the missing information was found in the mixed methods category when it came to the question of discrepancies and contradictions between quantitative and qualitative results. The frequent rating of “Can’t tell” was also noticeable because no information about the category could be found in the articles.

**Table 1:** Reporting the results of the MMAT tool (Mixed Methods Studies)

Studies	1.1	1.2	1.3	1.4	1.5	4.1	4.2	4.3	4.4	4.5	5.1	5.2	5.3	5.4	5.5
Abe et al. (2023) [91]	X	X	X	X	X	?	/	X	/	X	X	X	X	?	X
Adepoju et al. (2022) [103]	X	X	X	X	X	?	/	X	?	X	X	X	X	?	X
Franke et al. (2021) [58]	X	X	X	X	X	X	?	X	X	X	X	X	X	?	X
Gadbois et al. (2022) [102]	X	X	X	X	X	X	/	X	?	X	X	X	X	?	X
Gusdal et al. (2023) [88]	X	X	X	X	X	X	?	X	/	X	X	X	X	/	X
Hansen et al. (2021) [54]	X	X	X	X	X	?	?	X	?	X	X	X	X	?	X
Hansen et al. (2024) [81]	X	X	X	X	X	X	/	X	/	X	X	X	X	/	X
Jansen-Kosterink et al. (2020) [131]	X	X	X	X	X	X	?	X	?	X	X	X	X	?	X
Johansson-Pajala et al. (2023) [89]	X	X	X	X	X	X	?	X	X	X	X	X	X	?	X
Kumar et al. (2023) [70]	X	X	X	X	X	X	?	X	/	X	X	X	X	/	X

^*^Categories 2 and 3 of the MMAT are not presented, as only quantitative descriptive studies were assessed with the MMAT

Most of the qualitative studies met the majority of the JBI tool criteria (Table 2). The primary deficiencies were related to the lack of cultural and theoretical positioning of the researchers and the reflection on the researchers' influence on the research.

**Table 2:** Reporting the results of the JBI tool (Qualitative Studies)

Studies	Criteria from the JBI Tool									
	1	2	3	4	5	6	7	8	9	10
Coll-Planas et al. (2021) [41]	X	X	X	X	X	X	X	X	X	X
Cryer et al. (2021) [34]	X	X	X	X	X	/	/	X	X	X
Hudson et al. (2020) [99]	X	X	X	X	X	/	/	X	X	X
Hudson et al. (2023) [86]	X	X	X	X	X	/	/	X	X	X
Leung et al. (2022) [97]	?	X	X	X	X	/	/	X	X	X
Mills et al. (2022) [42]	X	X	X	X	X	/	/	X	X	X

### Results of individual studies

Details about the results of the individual studies can be found in the Data Extraction Table (Additional file 7 Table 5: Data extraction table). The majority of the included studies reported loneliness as an outcome (77 studies), followed by social support (10 studies) and social isolation (7 studies). Various other social components, such as social network, social participation or social connection were described as additional outcomes. Significant improvements in relevant outcome parameters were reported in 49 studies following the interventions. In contrast, 14 studies reported non-significant differences, and 16 studies reported no exact findings compared to the control group or pre- and posttest.

The following description of the results of individual studies is structured based on the categorization of the interventions, which includes differentiation between analogue interventions, technological interventions and multicomponent (analogue and technological) interventions (Additional file 8 Table 6: Categorization system). Most interventions were conducted regularly, either once or twice per week. The duration of the interventions typically ranged from 4 weeks to 6 months. Follow-up periods were reported in 21 studies, lasting from 1 to 12 months.

### Analogue interventions

#### Individual interventions

Individual interventions were further divided into trainer-administered and self-administered approaches. Five studies were accompanied by a trainer [34–38]. The first study reported a reduction in social isolation following the implementation of a pet support program (PSP), which assisted the elderly in caring for their pets [34]. The effects of an integrated yoga therapy approach were investigated in an interventional study. Significant decreases in loneliness were observed after three months of regular daily practice sessions [38]. Another study provided a psychosocial intervention program (Acompaña-Té) for older women in Spain. This program, based on the cognitive model of loneliness, included conversations, attribution retraining, and behavioral activation. Following the intervention, a slight increase in the loneliness scores of both the intervention and control groups was observed, but this was not statistically significant [35]. In contrast, a randomized controlled trial examining the impact of a meditation program suggested that weekly meditation classes were an effective solution for alleviating loneliness in older adults [36]. Positive results were also reported for a wellness coaching program, which led to a significant decrease in loneliness after the intervention [37].

Two interventions were conducted in a self-administered manner without the participation of trainers [39, 40]. The first study reported significantly reduced loneliness following a self-affirmation intervention in the intervention group [39]. The second intervention investigated the effects of receiving letters from nursing students on perceived loneliness of older adults in faith communities [40]. Results demonstrated a significant decrease in the level of loneliness following the intervention.

#### Community interventions

Community interventions are structured programs and activities implemented within groups of three or more individuals, aimed at reducing feelings of loneliness and promoting social connections. Community interventions (16 studies) were further divided into group meetings (6 studies), educational and psychological interventions (2 studies), art and music interventions (6 studies), dance interventions (1 study) and religious interventions (1 study).

Group meetings included various types of activities and interactions. The first study evaluated a program promoting social capital among older people in primary health care, after which most participants reported reductions in loneliness in subsequent interviews [41]. Another qualitative study highlighted the effectiveness of an innovative aging-in-place model (Oasis Senior Supportive Living) in alleviating loneliness and improving quality of life [42]. One quasi-experimental study evaluated the effectiveness of a life board game centered around the themes of “thanks”, “sorry”, “love” and “farewell” in reducing loneliness among older adults [43]. The intervention group exhibited significantly larger improvements in loneliness scores compared to the control group. Another pilot study investigated the influence of a storytelling board game called “Kioku” on social connections and cognitive engagement [44]. While loneliness increased in the control group, a decrease was observed for the intervention group after 12 weeks. Additionally, a randomized controlled trial conducted in China employed a reminiscence therapy-based hybrid board game, which also resulted in significant reductions in loneliness levels post-intervention [45]. Similar results were reported in a study exploring the efficacy of group reminiscence therapy based on Chinese traditional festival activities [46].

Two studies were focusing on educational and psychological interventions to combat loneliness [47, 48]. The first study provided a psychosocial care model in an elderly care center, which led to a statistically significant reduction in loneliness scores after the intervention [47]. The second study implemented a social participation training program for older adults and reported a significant decrease in loneliness and an increase in social participation following the intervention [48].

Music interventions were examined in two studies [49, 50]. A cluster-randomized trial observed reductions in loneliness following weekly choir sessions [49]. Another study on a singing group program (SGP) also reported significant improvements in social well-being and a significant decrease in perceived loneliness [50]. However, these effects did not persist during the 6-month follow-up period. Similar positive effects on loneliness and social connectedness were noted in an intergenerational arts and heritage-based intervention [51]. Furthermore, a decrease in loneliness was observed following an art-based intervention. Another intervention highlighted the benefits of group art therapy in reducing the sense of loneliness and helplessness [52]. The last study in this subcategory investigated the effects of a weekly art-based museum intervention on perceived loneliness among older people and reported lower levels of loneliness for the intervention group [53].

One study implemented a dance intervention for older adults [54]. In contrast to the results of art and music interventions, a statistically increase in overall loneliness was observed following the dance intervention.

The last study in this category reported reductions for the sense of loneliness after a religious intervention based on Quran verses and narrations of infallible [55].

#### Mixed analogue interventions

Mixed analogue interventions were conducted in the context of 12 studies, differentiating between trainer-administered interventions (7 studies), courses on the use of technical devices (4 studies) and group reminiscence therapy in combination with physical exercises (1 study).

The first intervention included the components communication, feelings of competence and self-control and participation in social activities. Following the intervention, no statistically significant differences were found in the loneliness and social isolation variables [56]. In contrast, a social prescribing model implemented in South Korea led to a significant decrease of loneliness and increase of social participation [57]. One study investigated the influence of a physical activity intervention (Choose to Move) on self-identified lonely versus non-lonely older adults. While no change in loneliness was reported for the non-lonely group, a significant decrease in loneliness was overserved immediately after the intervention in participants who identified as lonely at baseline [58]. However, these results did not persist during the 12-month follow-up period, as described in the corresponding follow-up study [59]. The 18-month follow-up even reported an increase in loneliness scores among older participants compared to baseline [60]. Another multicomponent intervention involved Primary Health Care nurses, family physicians, social workers and neighborhood community agents. This intervention consisted of health promotion and disease prevention and activities aimed at improving the mental the emotional state and creating a social network among participants. Significant improvements of loneliness and social isolation were reported for the intervention group [61]. Similarly, one study evaluated the effect of a combination of group-based and person-centered interventions for women on loneliness, social support and social inclusion and observed significant benefits in all these outcomes [62].

Furthermore, analogue courses on the use of digital devices were implemented for older adults in four studies [63–66]. The first study provided a series of workshops covering the basic features of a tablet, which did not lead to any differences in social isolation, loneliness, or social support [64]. Ambiguous results were reported for an intervention that included training on the use of social network sites [65]. Although no significant differences were observed on the loneliness scale, participants reported a significant reduction in feelings of being left out. Another quasi-experimental study provided an internet information station with various lessons [63]. The intervention led to improved feelings of connectedness, though these improvements were not statistically significant. The final study in this subcategory observed significant improvements in perceived loneliness in both the intervention and the waitlist control group following a four-week social media training workshop [66].

For an intervention providing group reminiscence therapy in combination with physical exercises, no difference in loneliness scores was observed during the follow-up period [67].

### Technological interventions

#### Phone interventions

Phone interventions, including phone calls between volunteers and older adults, were conducted in three studies [68–70]. No significant changes in loneliness scores were observed in the first study following variable numbers of phone calls between students and older adults in the community [68]. In contrast, an intergenerational friendly telephone visit program, which included weekly calls, led to a significant decrease in loneliness following the intervention [70]. Similar results were reported for an intervention combining a loneliness helpline with regular telephone communication between older adults and their assigned trained volunteers to create new friendships [69].

#### Videoconferencing interventions

The majority of technological interventions were videoconferencing interventions (12 studies). This category was further divided into five subgroups, differentiating between synchronous and asynchronous, as well as individual and group interventions.

Synchronous individual interventions were conducted in two studies [71, 72]. The multi-faceted intervention in the first study included cognitive, affective, and behavioral components [71], while the second study focused on techniques derived from cognitive behavioral therapy and resilience training [72]. Both interventions resulted in reductions in perceived stress and loneliness. In the first study, these effects did not persist during the follow-up period, during which no meaningful changes in loneliness scores were observed [71].

One study described an asynchronous individual intervention involving instructional videos, discussions, and learning activities aimed at promoting goal-setting and problem-solving strategies [73]. No significant decrease in loneliness was measured following this intervention.

Ambiguous results were reported for synchronous group interventions. One study observed a significant decrease in social isolation after a 3-month cycle of weekly virtual museum tours [74]. Additionally, significant reductions in loneliness were reported following a videoconferencing-implemented program that included sports, skill development, and interactive social sciences courses [75]. Similar results were observed immediately after a short-term digital group intervention on Zoom [76]. These positive effects on loneliness were not maintained at follow-up one month later [77]. Another study in this subcategory investigated the benefits of a digital bidirectional compared to a unidirectional remote interaction, finding no significant differences in loneliness scores between both groups [78].

Synchronous exercise interventions were conducted in three studies. One case study reported positive changes in loneliness scores after virtually mentally stimulating activities over four weeks [79]. No significant changes in loneliness were found within a randomized controlled study investigating the effects of a synchronous tele-exercise program [80]. Nevertheless, the intervention was considered to positively contribute to coping with loneliness and improving mood. Quantitative data from a 12-week dance training intervention did not show significant effects on loneliness and social isolation [81]. In contrast, qualitative data suggested that implementing online dancing sessions may positively influence participants’ social engagement. Another study with a multidimensional design tested the effect of a social and cognitive online training (SCOT) on loneliness. Compared to a non-specific cognitive training group (CON), no significant differences were reported [82].

#### Web-based interventions

Five studies implemented web-based individual interventions [83–87]. The first study provided a web-based eHealth service aimed at motivating older adults to improve their eating behavior and decrease loneliness [83]. This intervention did not lead to a decrease in loneliness or differences in quality of life. Similarly, no differences in loneliness between the intervention and control groups were observed on the internet-based program “NümEinsam”, developed in Switzerland [87].

Significant reductions in loneliness were reported in the context of a mental health and psychosocial support program [84]. Further positive results were reported for an integrated Loneliness Alleviation Program (LAP) provided via YouTube [85]. The participants of a web-based loneliness intervention in the last study of this subcategory reported alleviated loneliness in semi-structured interviews following the program [86].

Web-based group interventions were conducted in three studies [88–90]. No reduction in experiences of loneliness was observed for a web platform for social interaction called “Fik@ room” (Fik from the word fika a social institution in Sweden) [88]. Another feasibility study examined the influence of this platform after 6-week and 12-week follow-up periods [89]. Although benefits on loneliness and social networks were observed after 6 weeks, these effects did not persist during the 12-week follow-up. In contrast, levels of perceived loneliness significantly decreased following an eight-week acceptance and commitment therapy (ACT) online intervention [90].

#### Application-based interventions

The effects of various digital applications on perceived loneliness of older adults were investigated in five studies [91–95]. The first tested the effects of a pedometer and communication application among older men, observing a decrease in loneliness in four out of seven participants, which was not considered significant [91]. In contrast, a mobile application developed to encourage social participation showed no significant changes in experienced loneliness [92]. Another study implementing the Personal Reminder Information and Social Management 2.0 (PRISM 2.0) software for older adults reported mixed results depending on the setting. Participants in rural locations and senior housing reported less loneliness and social isolation, whereas those in assisted living communities experienced no change in loneliness [95]. Additionally, one study investigated the impacts of Immersive Virtual Reality (IVR) on physical and mental dimensions of older adults and reported significant reductions in loneliness following the intervention [94].

#### AI-based interventions

One study aimed to test the use of AI in interventions against loneliness in older adults, utilizing the Amazon Alexa Echo device [96]. The study focused on the influence of the personal voice assistant on adults aged 75 years and older, with participants reporting significantly lower loneliness after the intervention [96].

#### Robot interventions

Robot interventions were further divided into three categories: humanoid robots (1 study) and pet robots (3 studies). One case study reported reduced feelings of loneliness after interaction with a humanoid social robot called KaKa [97]. Studies investigating the influence of pet robots reported similar results following the intervention. Quantitative data of the first study indicated that loneliness decreased with the help of an animatronic pet [98]. Based on semi-structured interviews, the second study suggested that robotic pets with interactive features were an effective solution for alleviating loneliness in older adults [99]. Another study provided interaction with a parrot-shaped robot, which hatched from an egg and grew into an adult robot. Statistically significant differences in loneliness scores between the intervention and control groups were observed after the intervention [100].

### Mixed technological interventions

Five studies were conducted using mixed technological interventions including several technical end devices, applications or online platforms. Two of them provided courses on the use of technical devices for older adults. Regarding the first pragmatic pilot trial, no changes in loneliness were found, although an improvement in perceived social support was observed after weekly digital training sessions [101]. The second novel pilot intervention, called “Talking Tech”, did not lead to significant quantitative differences in loneliness scales, but participants reported several benefits in reducing loneliness and social isolation [102]. One study paired older adults with college students for regular virtual interaction. This befriending intervention resulted in reductions in perceived loneliness [103]. Another study tested a combination of conversation and exercises in weekly Zoom or telephone calls. While the UCLA-20 scale demonstrated statistically significant pre-post differences, the UCLA-3 scale did not [104]. The last study used a multimodal approach based on a videocall system, including sessions with social workers, psychologists, sociocultural entertainers and physiotherapists [105]. Significant reductions in perceived loneliness were reported after participating in the virtual social center VERA for six months.

### Multicomponent (analogue and technological) interventions

Six studies implemented interventions combining analogue and technological approaches. Due to the heterogeneity of these interventions, this category was further divided based on the specific interventions described.

The first study investigated the effects of combining 3D virtual reality with hands-on horticultural activities, which resulted in significant improvements in isolation after the intervention [106]. Another befriending intervention between volunteers and older adults included various types of interaction such as chatting, arts, physical activities, and cognitive activities [107]. Loneliness was reduced, and loneliness literacy improved following the intervention. Healthcare services were implemented in two studies to reduce perceived loneliness [108, 109]. The community-based integrated service model, which combined eight healthcare services with five social care services in South Korea led to significant improvements in loneliness and social support [108]. Similar results were reported for another informal home care support intervention program (HoSIP) in South Korea, with significant relationships observed between the outcome variables loneliness, social network, and social support [109]. One study investigated the effects of a web-based intervention combined with physical activities, comparing a web-based and print-based intervention group with a wait-list control group. Although improvements were described for both the web-based and print-based interventions, these were not significant compared with the control group [110]. The last study in this category examined the influence of the Amazon Alexa Echo device combined with additional group meetings on loneliness and social network [111]. Positive effects on both outcomes were observed in this interventional study.

### Key findings

In summary, a trend towards technological interventions has been observed in recent years as a consequence of the coronavirus pandemic and the general advanced application and acceptance of technological solutions. Nonetheless, the results suggest that the efficacy of analogue interventions tends to be superior to that of technological interventions. Among analogue interventions, community interventions were particularly effective in reducing loneliness. For both analogue and technological interventions, it was found that courses on the use of technical devices contributed only to a limited extent to a reduction of loneliness. Inconsistent results were observed in the category of technological interventions, particularly with videoconferencing, web-based, and application-based interventions. In contrast, AI-based and robot interventions reported exclusively positive results in terms of reducing loneliness and social isolation. However, these results are not based on RCTs but mainly on observational studies and case reports.

## Discussion

### Main results

This systematic review aimed to provide a comprehensive and up—to-date overview of existing studies to identify interventions to combat loneliness among adults aged 60 years and older, regardless of specific diseases and comorbidities. The study identified a variety of interventions aimed at reducing loneliness across different populations and settings. Due to the heterogeneity of the interventions conducted, a categorization system was established to structure the results and examine the efficacy of the different categories. In summary, no specific form of intervention was shown to be unequivocally effective in preventing and reducing loneliness. Nevertheless, trends and tendencies for the various intervention categories will be discussed.

Earlier systematic reviews have already examined the efficacy of interventions targeting loneliness among older adults [18, 19, 22]. However, many of these studies have highlighted substantial methodological limitations, including small sample sizes, heterogeneous outcome measures, and limited use of randomized controlled designs. By focusing on studies published between 2020 and 2024, the present review provides an updated perspective on how interventions have evolved in recent years, particularly in the context of the COVID-19 pandemic and the increasing implementation of technological approaches.

For the period between 2020 and 2024, it can be stated that technological interventions have been increasingly implemented and their efficacy investigated. The trend towards technological interventions aligns with observations from previous publications, and it can be assumed that the coronavirus pandemic has further intensified this trend [112, 113]. Recent publications have reported mixed results regarding the efficacy of technological interventions [22, 23, 113]. These are consistent with the findings of this systematic review. Interestingly, all interventions considering Artificial Intelligence (AI) and robot interventions show significant improvements in loneliness. However, several methodological limitations should be taken into account when interpreting these findings. Randomization was frequently not performed, sample sizes were often small, and pre-registration of study protocols was lacking. These aspects may limit transparency and reduce the robustness of the reported effects. In addition, potential novelty effects and the increased attention associated with technology-based interventions may have influenced participants’ responses. Therefore, a positive publication bias for AI-based and robot interventions cannot be ruled out completely. As significant changes and developments are expected in the development of AI and robots in the coming years, these forms of intervention should also be considered in future interventions.

Despite the increase in technological interventions, this study found that analogue interventions tended to be superior in terms of their effectiveness. The direct interpersonal contact in face-to-face interactions could positively impact loneliness and social isolation [78, 114]. Furthermore, adults aged 80 years and older may be less familiar and practiced with using digital devices, which could limit the efficacy of technological interventions [115, 116]. In addition, technology interventions can present challenges, especially for older people with hearing or visual impairments, limited prior technology experience or people who live in rural areas with a poor internet connection [81].

Regarding analogue interventions against loneliness, community interventions appear to reliably contribute to reducing loneliness. Positive results were particularly noted in subcategories such as group meetings, educational and psychological interventions, and art and music interventions. In relation to the number of interventions examined in both subcategories, our study provides evidence that group interventions tend to be more effective than individual interventions. It is also conceivable that there was a selection bias, as people who are confident and want to actively work on their loneliness are more likely to have participated. Lonely people who live in seclusion are certainly less representative of the study population. In addition, many studies have used convenience sampling, which may increase the effect [48, 61, 64, 79]. Moreover, the participants were predominantly female, which could also have contributed to a selection bias [88, 92]. Future studies should address the question, whether there is a barrier to participate at an intervention against loneliness, if it is label as such due to stigmatization.

Benefits of community interventions compared to individual interventions have also been reported in earlier studies [18, 23, 112, 113, 117–119]. In contrast, group interventions were not classified as necessarily beneficial in other reviews [19, 22]. These contradictory results could be explained by the considerable heterogeneity of the group interventions in terms of their context and content. In addition, the comparatively favourable outcomes observed in several group-based interventions may be interpreted in light of established theoretical frameworks. Social support theory suggests that stable interpersonal relationships can buffer stress and enhance perceived belonging and social connectedness [120]. Group settings may therefore facilitate the development of sustainable support networks that extend beyond the duration of the intervention. Furthermore, the concept of active aging emphasizes continued participation, autonomy, and engagement in social and community life [121]. Group-based interventions may promote these dimensions by encouraging older adults to assume active roles within a shared social context, thereby strengthening perceived agency and social connectedness.

The findings on the effectiveness of multicomponent interventions are consistent with previous research [22, 119]. Consequently, further multicomponent interventions should be designed and included in future research.

Furthermore, numerous participants benefited from a combination of analogue and technological interventions. In a mixed intervention, the respective advantages of analogue and digital intervention can be taken advantage of. This includes the opportunity to reach a wide range of people in different regions with different individual needs.

In comparison with earlier systematic reviews, it seems that no interventions involving real animals have been conducted since 2020. Instead, research during this period has focused on examining the relationship between pet ownership, loneliness and social isolation [122, 123]. The findings suggest a strengthening of this relationship during the pandemic [122, 123]. However, previous studies have demonstrated the efficacy of animal interventions in combating loneliness and social isolation [22, 124]. One review highlighted positive outcomes for loneliness following animal therapy interventions [22]. Another review reported significant improvements in mental health due to animal-assisted interventions [124]. This recent neglect of animal interventions could be attributed to the circumstances of the coronavirus pandemic or the spirit of the time hyping technological alternatives such as animal robots more.

The efficacy of interventions targeting loneliness does not depend exclusively on the category of intervention. It has been emphasized that there is no "one-size-fits-all" intervention for effectively reducing loneliness [19, 92]. The individuality of the experience of loneliness may also affect the implementation of standardized interventions [19]. Previous studies emphasized that even the awareness of receiving treatment to reduce loneliness could influence participants’ social behavior [72, 113]. To reduce loneliness in the long term, the practices and behaviors learned as part of the interventions must be sustainably integrated into one's lifestyle [113]. However, only a minority of the included studies reported follow-up assessments, which ranged from one to twelve months, and several interventions showed diminishing effects over time. This may indicate that some interventions provide social interaction primarily during the intervention phase without establishing lasting social networks or routines that persist beyond the program. Future studies might therefore need to take participants’ preferences more strongly into account when designing interventions that aim to achieve sustainable effects.

The potential for bias was classified as high for the majority of the studies. This is partly due to the inherent difficulties of a scientific approach to loneliness and social isolation. One challenge is an adequate operationalization of these two concepts, which is reflected in the heterogeneity of the measurement instruments used. For example, a measurement of loneliness may incorrectly measure people's social isolation because the terms are used interchangeably. Although the UCLA Loneliness Scale was the predominant instrument used across studies and is considered a well-established and reliable measure, it primarily captures the subjective experience of loneliness. It remains debatable to what extent this instrument fully reflects the multidimensional nature of loneliness, which may encompass both emotional and social components. A more differentiated assessment of these dimensions may allow for a more nuanced evaluation of intervention effects.

In addition, the assessment of risk of bias in studies is often very poor, as the primary outcome, such as loneliness, was based on self-assessment instruments. There was a lack of blinding or external assessment of the outcome parameters. Although these scales have good reliability, the results may not fully represent the actual state of the participants [78]. Given that loneliness represents a subjective experience, the reliance on self-reported instruments may be particularly susceptible to reporting bias and social desirability effects. These aspects should be carefully considered when interpreting the reported outcomes. The sub-areas of the risk of bias tools should therefore be considered when assessing the quality of the studies.

Because patients were not blinded in most of the studies, participants were aware of their allocation, which may have influenced their responses. In this context, intervention studies targeting loneliness may also be prone to the Hawthorne effect. Participants may report improvements due to increased attention, enhanced social contact, or perceived expectations associated with study participation rather than the specific components of the intervention itself. However, blinding can be achieved by implementing modified interventions for the control group, as demonstrated in certain studies [39, 67, 78, 95]. Furthermore, numerous studies were conducted without a control group. Even when a control group was present, randomization was often not performed [35, 69, 108]. Significant differences in perceived loneliness within the samples at the beginning of the interventions were also noted. In some studies, loneliness or social isolation were explicitly defined as inclusion criteria. Consequently, when interpreting the results, it is necessary to differentiate between interventions aimed at preventing versus reducing loneliness.

The sample size in many of the studies was comparatively small, partly due to numerous dropouts [83, 103, 125]. Both the duration of the interventions themselves and the follow-up periods were with only some months quite short in most studies [76, 80].

Due to the methodological heterogeneity of study designs and outcome measures, sensitivity analyses were not feasible. Nevertheless, risk of bias was systematically assessed using design-specific appraisal tools and was considered in the narrative synthesis and interpretation of the findings.

In general, it must be noted that some of the study interventions and outcomes were significantly influenced by the coronavirus pandemic. In some studies, loneliness prevalence increased due to the pandemic, and no significant differences were found as a result of the intervention [78, 83, 95]. Additionally, the COVID-19 restrictions, affected participant diversity and sample size [126].

Furthermore, numerous confounders must be considered in the context of both concepts [22, 127, 128]. For example, loneliness is associated with increased healthcare utilization such as more frequent physician visits and increased impatient care [128]. Only a small number of studies have adequately addressed and reflected confounding factors.

### Future implications

For an adequate presentation and interpretation of the results, it is essential to clearly distinguish between the different outcomes of the studies. Numerous earlier publications have emphasized the need for a uniform definition of the concepts of loneliness and social isolation [18, 19, 127]. However, this literature review has revealed that even recent publications lack sufficiently standardized definitions. Consistency in defining terms is necessary to enhance accuracy, improve reporting and facilitating the replication of interventions across different contexts [19]. Consequently, future research should aim to establish a consensual definition and consistent use of the terms. E.g. there is a potential approach to appropriately address the heterogeneous definitions of loneliness as a subjective state resulting from a deficit in social relationships, which is perceived as unpleasant [129]. A corresponding interdisciplinary discussion regarding the various definitions should also be conducted internationally.

Regarding methodology, it is desirable to employ study designs with the presence of a control group, ideally under randomization [21, 112].

Furthermore, it is essential to harmonize and standardize instruments for measuring loneliness and social isolation to facilitate comparison of studies and synthesis of findings [130]. In addition, future research should further examine the broader implications of reductions in loneliness. While several interventions demonstrated statistically significant improvements in loneliness scores, it remains insufficiently explored to what extent these changes translate into sustained improvements in overall well-being. Given the established associations between loneliness and mental as well as physical health outcomes, future studies should assess whether intervention-related reductions in loneliness are accompanied by measurable benefits in psychological functioning, quality of life, and physical health. The efficacy of interventions also depends on the duration of the intervention and follow-up periods [20, 113]. However, the duration of an intervention as well as the proper “maintenance therapy” interval should be the matter of future research.

Future research should also aim to better differentiate the circumstances under which loneliness interventions are effective. Important contextual variables may include cultural background, geographical setting (e.g., rural versus urban environments), and the presence of pre-existing physical or mental health conditions. Several intervention studies have already been implemented in specific geographical and socioeconomic contexts, providing insights into the feasibility of loneliness interventions in real-world settings. For example, group reminiscence therapy among rural older adults living alone has shown promising effects on loneliness and perceived stress [46]. Other interventions have been integrated into existing healthcare and community structures, such as primary care–based programs promoting social participation or social prescribing initiatives [41, 57]. In addition, community-based programs such as the “Choose to Move” initiative illustrate the potential scalability of loneliness-related interventions beyond pilot settings [58–60]. Future research should further explore how such approaches can be adapted and implemented across diverse socioeconomic and geographical settings.

Interestingly, no study could be found that included an intervention where older people could share their skills and expertise on specific topics with others. There is a need for research in this area, especially as this form of intervention has significant potential to effectively reduce loneliness in different settings due to the individuality and variety of possible activities.

### Limitations

This systematic review has several strengths. It focuses on the concepts of isolation and loneliness as well as social support and social integration. A complex search strategy was employed to comprehensively search multiple databases for eligible studies. Furthermore, study screening and risk of bias assessment were conducted by two independent researchers. This is one of the first reviews to address AI and robotic interventions.

When searching for appropriate studies, it is possible that some studies were not indexed in the databases searched or were missing for other reasons. Additionally, only English and German language articles were included, which may have led to the exclusion of relevant research conducted in other languages. As a result, the findings may not fully reflect global perspectives on interventions against loneliness. Future reviews could consider broader language inclusion criteria to enhance international representativeness. Further limitations arise from the defined exclusion criteria, which were based on the setting, participants' health status, and level of care. The exclusion of institutional settings, such as nursing homes and assisted living facilities, enabled a focused analysis of interventions targeting community-dwelling older adults. However, this decision limits the generalizability of the findings to institutionalized populations, who may experience loneliness within distinct structural and social care contexts. This may have led to the omission of some relevant forms of intervention against loneliness. Due to the heterogeneity in outcome parameters, assessments, study populations, study designs and effect estimates among the studies, there was no intention to combine results statistically. Consequently, our findings on efficacy and effectiveness should be interpreted with caution.

## Conclusion

This systematic review identified that analogue interventions, particularly community interventions such as group meetings and educational or psychological interventions, are effective in alleviating loneliness. Technological interventions, especially those based on AI and robots, also demonstrated benefits in reducing perceived loneliness, however with some risks of bias. In contrast, heterogeneous results were reported for videoconferencing and web-based interventions. The combination of analogue and technological interventions in particular produced promising results regarding a decrease of loneliness. Specific interventions must be tailored to the target group and setting and regularly reevaluated. In order to improve the quality of healthcare for those affected, it is important to integrate approved and effective measures and behaviors into people's lifestyles and communities in a lasting and sustainable way. Future research should focus on differentiating the circumstances under which various forms of intervention are effective. The next steps should include to implement these interventions comprehensively and monitor their effectiveness over several years.

## Supplementary Information

Additional file 1: Table 1: PRISMA Checklist

Additional file 2: Table 2: Eligibility criteria

Additional file 3: Table 3: Search strategy

Additional file 4: Table 4: Outcome parameters

Additional file 5: Figure 1: RoB2 Traffic light plot

Additional file 6: Figure 2: ROBINS-I Traffic light plot

Additional file 7: Table 5: Data extraction table

Additional file 8: Table 6: Categorization system

## Data Availability

All data generated or analyzed during this study are included in this published article [and its supplementary information files.

## Abbreviations

AI: Artificial Intelligence
ACT: Acceptance and Commitment Therapy
ICT: Information and Communication Technology
JBI: Joanna Briggs Institute
MMAT: Mixed Methods Appraisal Tool
PICO: Population, Intervention, Control, Outcome
RCT: Randomized Controlled Trial
UCLA Loneliness Scale: University of California Loneliness Scale
