# Supplementary material for: Interventions against loneliness and social isolation in older adults– a systematic review
Source: BMC Public Health. 2026 May 18;26:1562. doi: 10.1186/s12889-026-27683-9 (PMC13182138; doi:10.1186/s12889-026-27683-9)
Supplement: Supplementary file 2 — Additional file 2: Table 2: Eligibility criteria [file 12889_2026_27683_MOESM2_ESM.docx]

Additional file 2 Table 2: Eligibility criteria

| PICOS | Inclusion criteria | Exclusion criteria |
| --- | --- | --- |
| Population | - Elderly (≥ 60 years old) who are affected or threatened by loneliness or social isolation | - People with specific diseases or comorbidities - Specific populations (e.g. immigrants, veterans, widows) - Pre-existing conditions (e.g. chronic conditions, physical or mental health) |
| Intervention | - Interventions against loneliness or social isolation |  |
| Control | - Standard care - Care as usual - Non-treatment - No active control |  |
| Outcome | - Reduced perceived loneliness or social isolation |  |
| Setting | - Outpatient care - Private Living | - Nursing homes / care homes / residential care / assisted living facilities - Interventions delivered in (community) hospitals |
| Design | - Languages: German or English - Retrospective and prospective longitudinal studies, randomised control trials, cross-sectional studies, reviews, meta-analyses, observational studies, case studies - Full text available | - Letters, comments, poster presentations, opinion, book reviews, editorials, methodological papers, abstracts, protocols, trial registrations |
| Timeframe | - Results from the last four years  (2020 – 2024) |  |
