# Supplementary material for: Interventions against loneliness and social isolation in older adults– a systematic review
Source: BMC Public Health. 2026 May 18;26:1562. doi: 10.1186/s12889-026-27683-9 (PMC13182138; doi:10.1186/s12889-026-27683-9)
Supplement: Supplementary file 3 — Additional file 3: Table 3: Search strategy [file 12889_2026_27683_MOESM3_ESM.docx]

Additional file 3 Table 3: Search strategy

| **MEDLINE**  = 3.327 results | **Scopus**  = 4.527 results | **CINAHL**  = 1.614 results | **Web of Science Core Collection**  = 4.594 results | **Cochrane Library**  = 661 results |
| --- | --- | --- | --- | --- |
| (elder*[TIAB] OR “elderly person*”[TIAB] OR “old people”[TIAB] OR “older people”[TIAB] OR “old adults”[TIAB] OR “older adults”[TIAB] OR “old individuals”[TIAB] OR “older individuals”[TIAB] OR pensioner*[TIAB] OR senior*[TIAB] OR “retired person*”[TIAB] OR retire*[TIAB] OR Retirement[MH])  AND  (intervention*[TIAB] OR “social intervention*”[TIAB] OR “psychosocial intervention*”[TIAB] OR “community based intervention*”[TIAB] OR “internet based intervention*”[TIAB] OR “social work”[TIAB] OR “social network*”[TIAB] OR “social service*”[TIAB] OR “social resource*”[TIAB] OR “social engagement”[TIAB] OR “social support”[TIAB] OR “social service agenc*”[TIAB] OR “social service provider*”[TIAB] OR “community resource*”[TIAB] OR “community health service*”[TIAB] OR “senior center*”[TIAB] OR “municipal health service*”[TIAB] OR “local health service*”[TIAB] OR “psychosocial support systems” OR “community support” OR “Senior Centers”[MH] OR “Social Work”[MH] OR “Psychosocial Intervention”[MH] OR “Internet-Based Intervention”[MH] OR “Social Networking”[MH] OR “Social Support”[MH] OR “Psychosocial Support Systems”[MH] OR “Community Support”[MH])  AND  (lone*[TIAB] OR “living alone”[TIAB] OR solitude[TIAB] OR solitary[TIAB] OR desolat*[TIAB] OR isolat*[TIAB] OR “social isolat*”[TIAB] OR “social exclu*”[TIAB] OR “social disconnect*”[TIAB] OR “social integrat*”[TIAB] OR “social participation”[TIAB] OR “social relat*”[TIAB] OR “social inclu*”[TIAB] OR “social wellbeing”[TIAB] OR “social cohesion”[TIAB] OR “social connect*”[TIAB] OR “sense of community”[TIAB] OR “community feeling”[TIAB] OR “community participation”[TIAB] OR “managing loneliness”[TIAB] OR Loneliness[MH] OR “Social Isolation”[MH] OR “Social Integration”[MH] OR “Social Cohesion”[MH] OR “Social Inclusion”[MH] OR “Social Participation”[MH]) | (TITLE-ABS-KEY(elder* OR {elderly person*} OR {old people} OR {older people} OR {old adults} OR {older adults} OR {old individuals} OR {older individuals} OR pensioner* OR senior* OR {retired person*} OR retire*))  AND  (TITLE-ABS-KEY(intervention* OR {social intervention*} OR {psychosocial intervention*} OR {community based intervention*} OR {internet based intervention*} OR {social work} OR {social network*} OR {social service*} OR {social resource*} OR {social engagement} OR {social support} OR {social service agenc*} OR {social service provider*} OR {community resource*} OR {community health service*} OR {senior center*} OR {municipal health service*} OR {local health service*} OR {psychosocial support systems} OR {community support}))  AND  (TITLE-ABS-KEY(lone* OR {living alone} OR solitude OR solitary OR desolat* OR isolat* OR {social isolat*} OR {social exclu*} OR {social disconnect*} OR {social integrat*} OR {social participation} OR {social relat*} OR {social inclu*} OR {social wellbeing} OR {social cohesion} OR {social connect*} OR {sense of community} OR {community feeling} OR {community participation} OR {managing loneliness})) | (TI elder* OR AB elder* OR TI “elderly person#” OR AB “elderly person#” OR TI “old people” OR AB “old people” OR TI “older people” OR AB “older people” OR TI “old adults” OR AB “old adults” OR TI “older adults” OR AB “older adults” OR TI “old individuals” OR AB “old individuals” OR TI “older individuals” OR AB “older individuals” OR TI pensioner# OR AB pensioner# OR TI senior# OR AB senior# OR TI “retired person#” OR AB “retired person#” OR TI “retired people” OR AB “retired people” OR TI retire* OR AB retire* OR MH Retirement)  AND  (TI intervention# OR AB intervention# OR TI “social intervention#” OR AB “social intervention#” OR TI “psychosocial intervention#” OR AB “psychosocial intervention#” OR TI “community based intervention#” OR AB “community based intervention#” OR TI “internet based intervention#” OR AB “internet based intervention#” OR TI “social work” OR AB “social work” OR TI “social network*” OR AB “social network*” OR TI “social service#” OR AB “social service#” OR TI “social resource#” OR AB “social resource#” OR TI “social engagement” OR AB “social engagement” OR TI “social support” OR AB “social support” OR TI “social service agencies” OR AB “social service agencies” OR TI “social service provider#” OR AB “social service provider#” OR TI “community resource#” OR AB “community resource#” OR TI “community health service#” OR AB “community health service#” OR TI “senior center#” OR AB “senior center#” OR TI “municipal health and care service#” OR AB “municipal health and care service#” OR TI “local health service#” OR AB “local health service#” OR TI “psychosocial support systems” OR AB “psychosocial support systems” OR TI “community support” OR AB “community support” OR MH “Senior Centers”+ OR MH “Social Work”+ OR MH “Psychosocial Intervention”+ OR MH “Internet-Based Intervention”+ OR MH “Social Networking”+ OR MH “Social Support”+ OR MH “Psychosocial Support Systems”+ OR MH “Community Support”+)  AND  (TI lone* OR AB lone* OR TI “living alone” OR AB “living alone” OR TI solitude OR AB solitude OR TI solitary OR AB solitary OR TI desolat* OR AB desolat* OR TI isolat* OR AB isolat* OR TI “social isolat*” OR AB “social isolat*” OR TI “social exclu*” OR AB “social exclu*” OR TI “social disconnect*” OR AB “social disconnect*” OR TI “social integrat*” OR AB “social integrat*” OR TI “social participation” OR AB “social participation” OR TI “social relat*” OR AB “social relat*” OR TI “social inclu*” OR AB “social inclu*” OR TI “social wellbeing” OR AB “social wellbeing” OR TI “social cohesion” OR AB “social cohesion” OR TI “social connect*” OR AB “social connect*” OR TI “sense of community” OR AB “sense of community” OR TI “community feeling” OR AB “community feeling” OR TI “community participation” OR AB “community participation” OR TI “managing loneliness” OR AB “managing loneliness” OR MH Loneliness+ OR MH “Social Isolation”+ OR MH “Social Integration”+ OR MH “Social Cohesion”+ OR MH “Social Inclusion”+ OR MH “Social Participation”+ OR MH “Community Programs”+) | (elder* OR “elderly person$” OR “old people” OR “older people” OR “old adults” OR “older adults” OR “old individuals” OR “older individuals” OR “pensioner$” OR “senior$” OR “retired person$” OR “retire*”)  AND  (intervention$ OR “social intervention$” OR “psychosocial intervention$” OR “community based intervention$” OR “internet based intervention$” OR “social work” OR “social network*” OR “social service$” OR “social resource$” OR “social engagement” OR “social support” OR “social service agenc*” OR “social service provider$” OR “community resource$” OR “community health service$” OR “senior center$” OR “municipal health service$” OR “local health service$” OR “psychosocial support systems” OR “community support”)  AND  (“lone*” OR “living alone” OR “solitude” OR “solitary” OR “desolat*” OR “isolat*” OR “social isolat*” OR “social exclu*” OR “social disconnect*” OR “social integrat*” OR “social participation” OR “social relat*” OR “social inclu*” OR “social wellbeing” OR “social cohesion” OR “social connect*” OR “sense of community” OR “community feeling” OR “community participation” OR “managing loneliness”) | (elder* OR “elderly person” OR “elderly persons” OR “old people” OR “older people” OR “old adults” OR “older adults” OR “old individuals” OR “older individuals” OR pensioner* OR senior* OR “retired person” OR “retired persons” OR retire*)  AND  (intervention* OR “social intervention” OR “social interventions” OR “psychosocial intervention” OR “psychosocial interventions” OR “community based intervention” OR “community based interventions” OR “internet based intervention” OR “internet based interventions” OR “social work” OR “social network” OR “social networks” OR “social networking” OR “social service” OR “social services” OR “social resource” OR “social resources” OR “social engagement” OR “social support” OR “social service agency” OR “social service agencies” OR “social service provider” OR “social service providers” OR “community resource” OR “community resources” OR “community health service” OR “community health services” OR “senior center” OR “senior centers” OR “municipal health service” OR “municipal health services” OR “local health service” OR “local health services” OR “psychosocial support systems” OR “community support”)  AND  (lone* OR “living alone” OR “solitude” OR “solitary” OR desolat* OR isolat* OR “social isolation” OR “social isolated” OR “social exclusion” OR “social excluded” OR “social disconnection” OR “social disconnected” OR “social integration” OR “social integrated” OR “social participation” OR “social relation” OR “social related” OR “social inclusion” OR “social included” OR “social wellbeing” OR “social cohesion” OR “social connection” OR “social connected” OR “sense of community” OR “community feeling” OR “community participation” OR “managing loneliness”) |
