# Supplementary material for: Interventions against loneliness and social isolation in older adults– a systematic review
Source: BMC Public Health. 2026 May 18;26:1562. doi: 10.1186/s12889-026-27683-9 (PMC13182138; doi:10.1186/s12889-026-27683-9)
Supplement: Supplementary file 4 — Additional file 4: Table 4: Outcome parameters [file 12889_2026_27683_MOESM4_ESM.docx]

Additional file 4 Table 4: Outcome parameters

| **Outcome parameter relevant to the results** | **Measurement** | **Studies (Authors)** |
| --- | --- | --- |
| **Loneliness** | UCLA Loneliness Scale (Russel et al., 1978) | Watson et al. (2023) |
|  | R-UCLA Loneliness Scale (Revised)  (Russell et al., 1980) | Mullins et al. (2020)  Kim et al. (2021)  Nazar et al. (2021)  Noh et al. (2021)  Dattilo et al. (2022)  Li et al. (2022)  Ae-Ri et al. (2023)  Dinet et al. (2023)  Liu et al. (2023)  Lim (2023)  Liu et al. (2024) |
|  | UCLA Loneliness Scale (Version 3)  (Russell et al., 1996) | Borji & Tarjoman (2020)  Esmaeilzadeh & Oz (2020)  Tkatch et al. (2020)  Aydin & Kutlu (2021)  Lorente-Martínez et al. (2021)  Quinn (2021)  Rodríguez-Romero et al. (2021)  Sandu et al. (2021)  Chen & Tsai (2022)  Jeste et al. (2022)  Gusdal et al. (2023)  Long (2023)  Panigrahi et al. (2023)  Johansson-Pajala et al. (2023)  Patapoff et al. (2023)  McKay et al. (2023)  Mierzwicki et al. (2023)  Czaja et al. (2024)  Hansen et al. (2024)  Kousha et al. (2024) |
|  | 3-item UCLA Loneliness Scale / UCLA Loneliness Scale (Version 3) (Russell et al., 1996) | Adepoju et al. (2022)  Balta et al. (2023) |
|  | Three-item version of the UCLA Loneliness Scale  (Hughes et al., 2004) | Fields et al. (2020)  Rolandi et al. (2020)  Franke et al. (2021)  Shapira et al. (2021a)  McKay et al. (2021)  Shapira et al. (2021b)  Weaver et al.  Hansen et al. (2021)  Gadbois et al. (2022)  Fullen et al. (2022)  Mierzwicki et al. (2023)  Zarling et al. (2023) |
|  | 9-item short version UCLA Loneliness Scale  (Luhmann et al., 2016) | Dworschak et al. (2024) |
|  | 8-item UCLA Loneliness Scale (ULS)  (Hays & DiMatteo, 1987; Zhou et al., 2012; Xu et al., 2018) | Ho et al. (2021)  Jones et al. (2021)  Ren et al. (2021)  Bodner et al. (2024)  Yan et al. (2024) |
|  | UCLA-6 item Loneliness Scale  (Wongpakaran et al., 2020) | Hsu et al. (2020) |
|  | UCLA Loneliness Scale short version – 4 item  (Pocinho et al., 2010) | Galinha et al. (2021) |
|  | UCLA Loneliness Scale short version – 4 item  (Adapted from Russel et al., 1980) | Dang et al. (2023) |
|  | NIH Toolbox for the Assessment of Neurological and Behavioral Function (Hodes et al., 2013) | Johnson et al. (2020) |
|  | Ando-Osada-Kodama Loneliness Scale (Ando et al., 2000) | Abe et al. (2023) |
|  | De Jong-Giervald Loneliness scale - six items  (De Jong-Gierveld & Kamphuls, 1985; De Jong-Gierveld & Van Tilburg, 2006; De Jong Gierveld & Tilburg, 2010) | Pandya (2021)  Hernández-Ascanio et al. (2021)  Neil-Sztramko et al. (2020)  Jansen-Kosterink et al. (2022)  Kramer et al. (2022)  Marliana et al. (2022)  Janssen et al. (2023) |
|  | Interviews and Surveys | Hudson et al. (2020)  Scherr et al. (2020)  Coll-Planas et al. (2021)  Hudson et al. (2023)  Janssen et al. (2023) |
|  | Loneliness Scale for the Elderly-LSE  (De Jong & Kamphuhls 1985; De Jong Gierveld & Van Tilburg, 1999) | Alpozgen et al. (2022)  Ilgaz & Gözüm (2023)  Yavuz & Şahin (2023) |
|  | ESTE scale (ESTE-R)  (Rubio & Aleixandre, 1999; Pinel-Zafra et al. 2010) | Palacios-Navarro et al. (2024)  Mouriño-Ruiz et al. (2024) |
|  | CES-D scale (Radloff, 1977) | Lippke et al. (2022) |
|  | PROMIS Social Isolation Scale − Short Form 8a (PROMIS) (National Academies of Sciences E, 2020) | Kumar et al. (2023) |
|  | Italian Social and Emotional Loneliness Scale (ISELS) (Zammuner, 2008) | Funghi et al. (2024) |
| **Number of social activities and contacts and satisfaction with these**  **Social network** | Social network questionnaire  (Larsson et al., 2013; Rudman et al. 2004) | Johansson-Pajala et al. (2023) |
|  | Six-item Lubben Social Network Scale (Jang et al., 2022) | Kousha et al. (2024) |
|  | Interviews | Scherr et al. (2020)  Leung et al. (2022)  Gadbois et al. (2022) |
| **Social isolation** | 11-item Duke Social Support Index (DSSI)  (Koenig et al., 1993) | Beauchet et al. (2022)  Hernández-Ascanio et al. (2021)  Neil-Sztramko et al. (2020) |
|  | Three items adapted from two questions focused on frequency of contact (Vernoff et al., 1957) | Franke et al. (2021)  McKay et al. (2023) |
|  | Interviews | Cryer et al. |
|  | Short-form scale of perceived isolation  (Cornwell & Waite, 2009) | Fan et al. (2022) |
| **Social participation** | Subjective Social Participation Index (SSPI) subscale of the ESTE II scale (Pinel et al., 2010) | Lorente-Martínez et al. (2021) |
|  | Social participation scale for the elderly living alone  (Cho & Yoo, 2016) | Kim et al. (2021) |
| **Social support** | ENRICHD (Enhancing Recovery in Coronary Heart Disease) ESSI (ENRICHD Social Support Instrument)  (Jeon et al., 2012) | Noh et al. (2021) |
|  | 12-item Multidimensional Scale of Perceived Social Support (Zimet et al., 1990) | Ilgaz & Gözüm (2023)  Yavuz & Şahin (2023)  Kousha et al. (2024) |
|  | 12-item Lubben Social Network Scale  (Lubben et al., 2006) | Neil-Sztramko et al. (2020) |
|  | Interpersonal Support Evaluation List (ISEL)  (Cohen & Hoberman, 1983) | Fields et al. (2020) |
|  | Duke-UNC-11 Functional Social Support Questionnaire (Bellon et al., 1996) | Rodríguez-Romero et al. (2021)  Shapira et al. (2021b) |
|  | MOS Social Support Survey (Ware & Sherbourne, 1992) | Czaja et al. (2024) |
|  | Inventory of Socially Supportive Behavior (ISSB)  (Barrera et al., 1981) | Liu et al. (2023) RCT |
| **Social connection** | Social connectedness scale  (Lee & Robbins, 1995) | Czaja et al. (2024)  Quinn (2021) |
| **Social Inclusion** | Social Inclusion Scale (Secker et al., 2009) | Ilgaz & Gözüm (2023) |
| **Social engagement** | 6-item Lubben Social Network Scale (LSNS-6)  (Lubben et al., 2006) | Rolandi et al. (2020) |
| **Social Well-Being** | Scale of Social Well-Being (Keyes, 1998) | Galinha et al. (2021) |
| **Social Identification** | Four Item Measure of Social Identification FISI  (Doosje, Spears, & Ellemers, 1995) | Galinha et al. (2021) |
| **Perceptions of social integration** | Social integration sub-scale of the social provisions scale (Cutrona & Russell, 1987) | Quinn (2021) |
| **Hopelessness** | The Beck Hopelessness Scale (BHS) (Beck et al., 1974) | Aydin & Kutlu (2021) |
| **Interpersonal Communication** | Interpersonal Communication Scale (ICS)  (Campbell & Atas Akdem, 2016) | Chen & Tsai (2022) |
| **Interpersonal Relationship** | Interpersonal Relationship Scale (Chien, 2004) | Chen & Tsai (2022) |
| **Influence on social network, health and well-being** | Interviews | Mills et al. (2022) |

**References**

Ando T, Osada H, Kodama Y. Construction of a new loneliness scale and correlates ofloneliness among middle aged and aged. Journal of the College of Education and HumanSciences, 2020; 3, 19–27.

Barrera M, JrSandler IN, Ramsay TB. Preliminary development of a scale of social support: Studies on college students. American Journal of Community Psychology, 1981; 9(4), 435–447. <https://doi.org/10.1007/BF00918174>

Beck AT, Weissman A, Lester D, Trexler L. The measurement of pessimism: The hopelessness scale. Journal of Consulting and Clinical Psychology, 1974; 42(6), 861–865.

Bellon JA, Delgado A, Luna J, Lardelli P. Validez y fiabilidad del cuestionario de apoyo social funcional Duke-Unc11. Atencion Primaria 1996; 18: 153–63

Campbell JM, Atas Akdemir O. The development of interpersonal communication scale: The study of validity and reliability. Electron. Turk. Stud. 2016; 11, 859–872.

Cho SH, Yoo YS. The effects of participation in social activity on life satisfaction in low-income aged people living alone: focusing on the mediating effects of loneliness. Korean Soc Gerontol Soc Welfare. 2016;71(4):35-59

Chien SCA. Comparison of Older Adults in Jia-yi Who Were Learning Participated and Nonparticipated in Terms of Their Interpersonal Relationships and Life Satisfaction. Master’s Thesis, National Chung Cheng University, Tainan County, Taiwan, 2004.

Cohen S, Hoberman HM. Positive events and social supports as buffers of life change stress. Journal of Applied Social Psychology, 1983; 13(2), 99–125.

Cornwell EY, Waite LJ. Social disconnectedness, perceived isolation, and health among older adults. J Health Soc Behav. 2009;50(1):31–48 PMID: 19413133.

Cutrona CE and Russell DW. The provisions of social relationships and adaptation to stress. (1987) In Jones WH and Perlman D (eds), Advances in Personal Relationships, Vol. 1. Greenwich, CT: JAI Press, pp. 37–67.

De Jong-Gierveld J, Kamphuls F. The Development of a Rasch-Type Loneliness Scale. Applied Psychological Measurement, 1985; 9(3), 289–299. <https://doi.org/10.1177/014662168500900307>

De Jong Gierveld J, Van Tilburg T. Manual of the loneliness scale. Dep Soc Res Methodol Vrije Univ Amsterdam, 1999.

De Jong-Gierveld J & Van Tilburg T. A 6-Item Scale for Overall, Emotional, and Social Loneliness. Research on Aging, 2006; 28(5), 582–598. https://doi.org/10.1177/0164027506289723

De Jong Gierveld J, Van Tilburg T. The De Jong Gierveld short scales for emotional and social loneliness: tested on data from 7 countries in the UN generations and gender surveys. Eur J Ageing 2010 Jun;7(2):121-130. <https://doi.org/10.1007/s10433-010-0144-6>

Doosje B, Ellemers N, Spears R. Perceived intragroup variability as a function of group status and identification. J Exp Soc Psychol. 1995;31:410–36

Hays RD, DiMatteo MR. A short-form measure of loneliness. J. Pers. Assess. 1987; 51, 69–81. doi: 10.1207/s15327752jpa5101_6

Hodes RJ, Insel TR, Landis SC. NIH Blueprint for Neuroscience Research. The NIH toolbox: Setting a standard for biomedical research. Neurology, 2013; 80 (11 Suppl. 3), S1. doi:10.1212/WNL.0b013e3182872e90

Hughes ME, Waite LJ, Hawkley LC, Cacioppo JT. A Short Scale for Measuring Loneliness in Large Surveys: Results From Two Population-Based Studies. Res Aging. 2004;26(6):655-672. doi:10.1177/0164027504268574

Jang Y, Powers DA, Park NS, Chiriboga DA, Chi I, Lubben J. Performance of an abbreviated Lubben Social Network Scale (LSNS-6) in three ethnic groups of older Asian Americans. Gerontologist 2022;62: e73-81

Jeon GS, Jang SN, Park SS. Social support, social network, and frailty in Korean elderly. J Korean Geriatr Soc. 2012;16(2):84–94. <https://doi.org/10.4235/jkgs.2012.16.2.84>.

Keyes CL. Social well-being. Soc Psychol Quart. 1998;61(2):121–40.

Koenig HG, Westlund RE, George LK, Hughes DC, Blazer DG, Hybels C. Abbreviating the duke social support index for use in chronically ill elderly individuals. Psychosomatics. (1993) 34:61–9. doi: 10.1016/S0033-3182(93)71928-3

Larsson E, Nilsson I, Larsson Lund M. Participation in social internet-based activities: five seniors’ interventionprocesses. Scand J Occup Ther. 2013;20(6):471–80. doi:10.3109/11038128.2013.839001.29.

Lee RM, Robbins SB. Measuring belongingness: the social connectedness and the social assurance scales. Journal of Counseling Psychology 1995; 42, 232–241

Lubben J, Blozik E, Gillmann G, Iliffe S, von RKW, Beck JC, et al. Performance of an abbreviated version of the Lubben Social Network Scale among three European community-dwelling older adult populations. Gerontologist 2006 Aug;46(4):503-513.

Luhmann M, Bohn J, Holtmann J, Koc, T, Eid, M. I’m lonely, can’t you tell? Convergent validity of self- and informant ratings of loneliness. J. Res. Pers. 2016; 61, 50–60. <https://doi.org/10.1016/j.jrp.2016.02.002>.

National Academies of Sciences E, Medicine: Social Isolation and Loneliness in Older Adults: Opportunities for the Health Care System. Washington, DC: National Academies Press (US), 2020

Pinel M, Rubio L, Rubio R. Un instrumento de medición de la soledad social: Escala ESTE II. 2010. Available from: http://envejecimiento.csic.es/documentos/documentos/rubio-soledad-este2.pdf

Pocinho M, Farate C, Dias CA. Validaç~ao psicométrica da escala UCLA-loneliness para idosos portugueses. [Psychometric validation of the UCLA-loneliness scale for Portuguese elders]. Interações, 2010; 10(18), 65–77.

Radloff LS. The CES-D Scale: a self-report depression scale for research in the general population. Applied Psychol Measure 1977;1(3):385-401. [doi: 10.1177/014662167700100306]

Rubio R, Aleixandre M. La escala “Este”, un indicador objetivo de soledad en la tercera edad. Geriatrika, Revista Iberoamericana De Geriatría y Gerontología 15 1999 ; 26–35.

Rudman A, Hutell D, Gustavsson P. Sjuksköterskors karriärvägar och hälsoutveckling de första åren efterutbildning. Enkät använd vid LUST-projektets datainsamling för X2004-kohorten fem år efter examen[Nurses’ career paths and health development in the first years after education. In: Questionnaire used in theLUST project’s data collection for the X2004 cohort five years after graduation].

Russell D, Peplau LA, Ferguson ML. Developing a measure of loneliness. J Pers Assess. 1978;42(3):290-294. doi:10.1207/s15327752jpa4203_11

Russell D, Peplau LA, Cutrona CE. The revised UCLA Loneliness Scale: concurrent and discriminant validity evidence. J Pers Soc Psychol. 1980;39(3):472-480. doi:10.1037//0022-3514.39.3.472

Russell DW. UCLA Loneliness Scale (Version 3): reliability, validity, and factor structure. J Pers Assess. 1996;66(1):20-40. doi:10.1207/s15327752jpa6601_2

Secker J, Hacking S, Kent L, Shenton J, Spandler H. 'Development of a measure of social inclusion for arts and mental health project participants', Journal of Mental Health. 2009; 18:1,65 — 72

Vernoff J, Kulka RA, EAM D. Mental health in America: Patterns of helpseeking from, 1957 to 1976. New York: Basic Books. p. 19

Ware JE Jr, Sherbourne CD. The MOS 36-item short-form health survey (SF-36). I. Conceptual framework and item selection. Med Care. 1992;30(6):473-483.

Wongpakaran N, Wongpakaran T, Pinyopornpanish M et al. Development and validation of a 6-item revised UCLA loneliness scale (RULS-6) using Rasch analysis. Br J Health Psychol 2020; 25: 233–256. <https://doi.org/10.1111/bjhp.12404>.

Xu S, Qiu D, Hahne J, Zhao M, Hu M. Psychometric properties of the short-form UCLA Loneliness Scale (ULS-8) among Chinese adolescents. Medicine 2018; 97, e12373.

Zammuner VL. Italians-social and emotional loneliness: the results of five studies. International Journal of Educational and Pedagogical Sciences, 2008; 2(4), 416–428

Zhou L, Li Z, Hu M, Xiao S. Zhong Nan Da Xue Xue Bao Yi Xue Ban. 2012;37(11):1124-1128. doi:10.3969/j.issn.1672-7347.2012.11.008

Zimet GD, Powell SS, Farley GK, Werkman S, Berkoff KA. Psy chometric characteristics of the multidimensional scale of per ceived social support. J Pers Assess 1990; 55: 610–617. https://doi.org/10.1080/00223891.1990.9674095
