# Supplementary figures and images for: Interventions against loneliness and social isolation in older adults– a systematic review
Source: BMC Public Health. 2026 May 18;26:1562. doi: 10.1186/s12889-026-27683-9 (PMC13182138; doi:10.1186/s12889-026-27683-9)

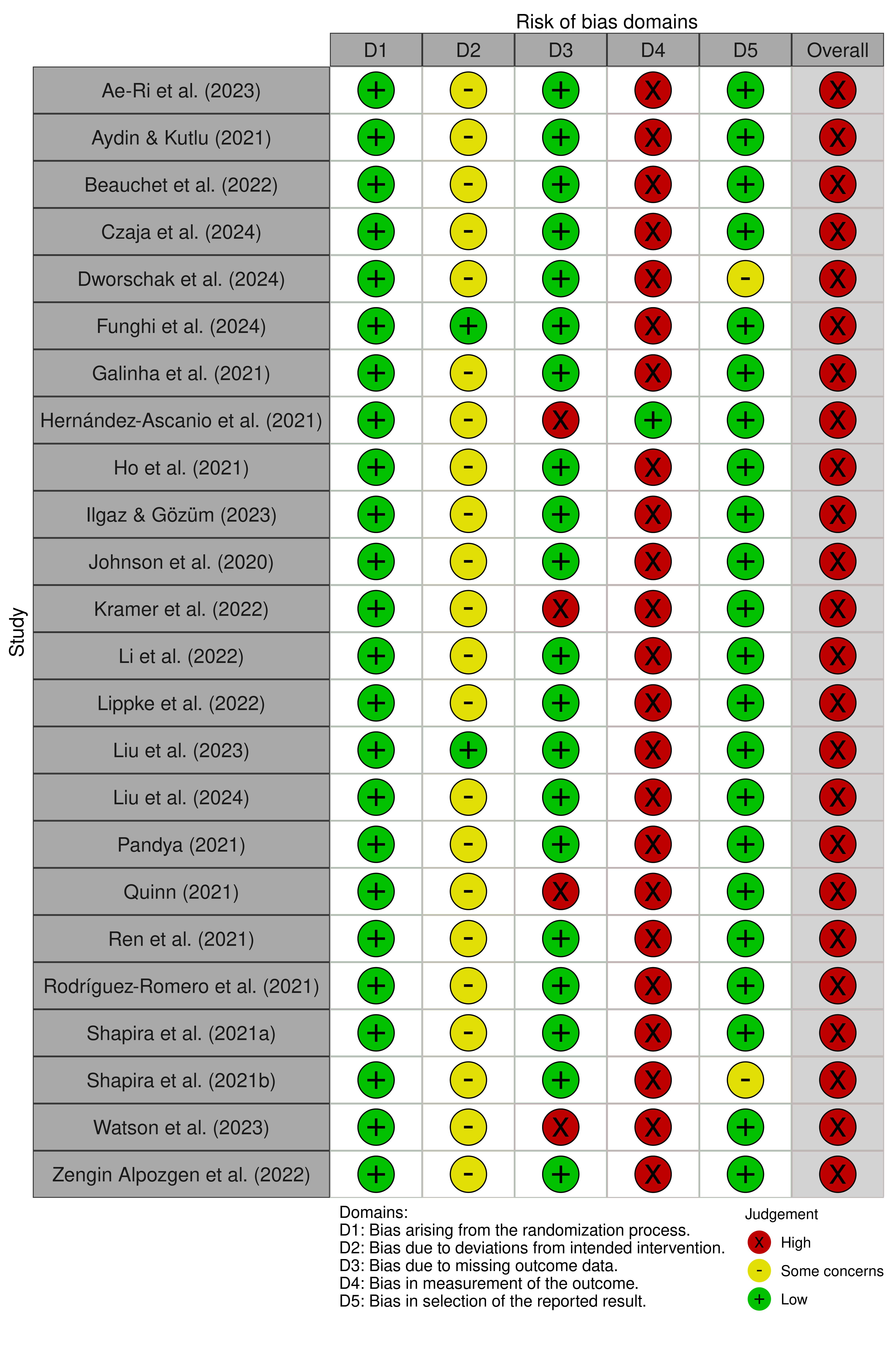

Supplement: Supplementary file 5 — Additional file 5: Figure 1: RoB2 Traffic light plot [file 12889_2026_27683_MOESM5_ESM.jpg]

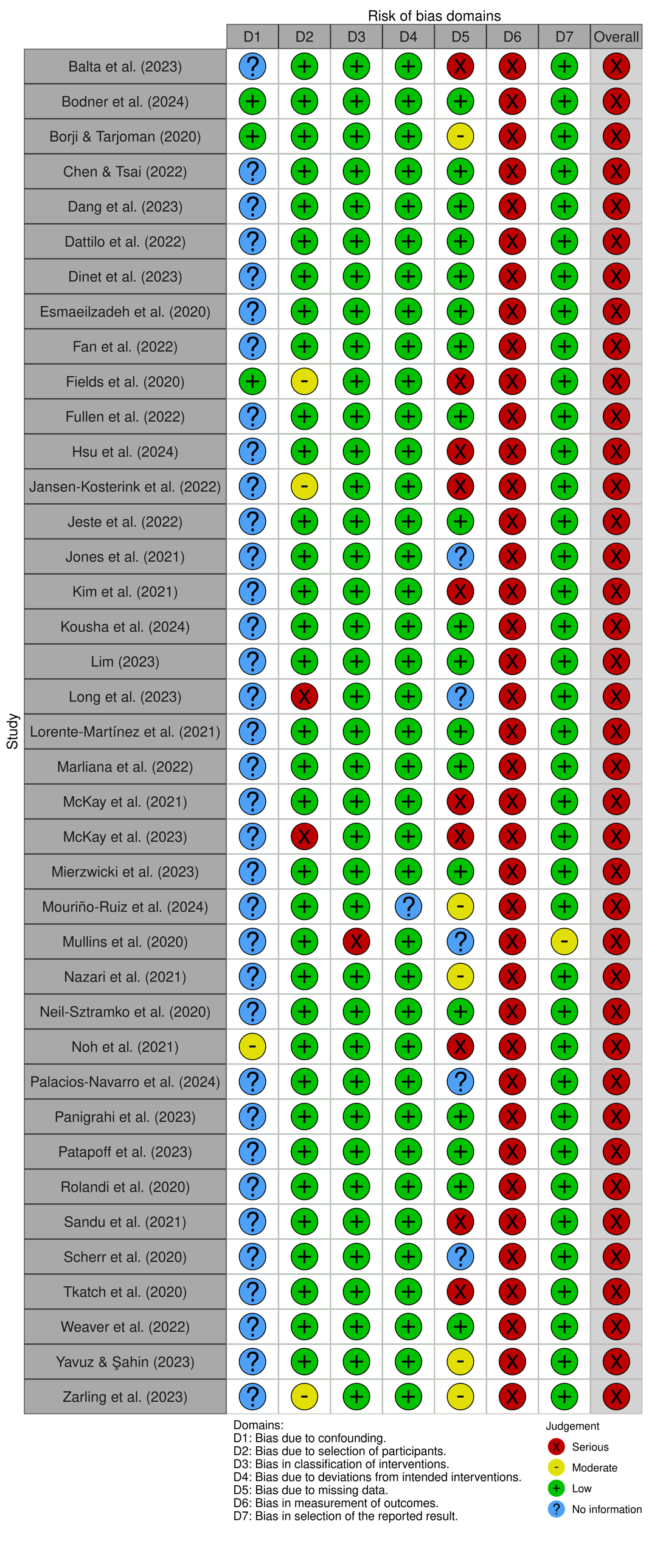

Supplement: Supplementary file 6 — Additional file 6: Figure 2: ROBINS-I Traffic light plot [file 12889_2026_27683_MOESM6_ESM.png]
