# Supplementary material for: Interventions against loneliness and social isolation in older adults– a systematic review
Source: BMC Public Health. 2026 May 18;26:1562. doi: 10.1186/s12889-026-27683-9 (PMC13182138; doi:10.1186/s12889-026-27683-9)
Supplement: Supplementary file 8 — Additional file 8: Table 6: Categorization system [file 12889_2026_27683_MOESM8_ESM.docx]

Additional file 8 Table 6: Categorization system

| **Interventions against loneliness** | | | | | | | | | | |
| --- | --- | --- | --- | --- | --- | --- | --- | --- | --- | --- |
| **Analogue interventions** | | | **Technological interventions** | | | | | |  | **Analogue and technological interventions** |
| Individual interventions (7) | Community interventions (16) | Mixed analogue  interventions (12) | Phone interventions (3) | Videoconferencing  interventions (12) | Web-based interventions (8) | Application-based interventions (5) | AI-based interventions (1) | Robot interventions (4) | Mixed technological interventions (5) | Multicomponent interventions (6) |
| a) Trainer-administered (5) Cryer et al. (2021) Lorente-Martínez et al. (2021) Pandya (2021) Fullen et al. (2022) Panigrahi et al. (2023) | a) Group meetings (6) Coll-Planas et al. (2021) Chen & Tsai (2022) Li et al. (2022) Mills et al. (2022) Bodner et al. (2024) Liu et al. (2024) | a) Trainer-administered (7) Hernández-Ascanio et al. (2021) Franke et al. (2021) Kim et al. (2021) McKay et al. (2021) McKay et al. (2023) Rodríguez-Romero et al. (2021) Ilgaz & Gözüm (2023) | Sandu et al. (2021) Balta et al. (2023) Kumar et al. (2023) | a) Synchronous individual interventions (2) Jeste et al. (2022) Patapoff et al. (2023) | a) Individual interventions (5) Kramer et al. (2022) Marliana et al. (2022) Ae-Ri et al. (2023) Hudson et al. (2023) Dworschak et al. (2024) | Abe et al. (2022) Jansen-Kosterink et al. (2022) Dinet et al. (2023) Janssen et al. (2023) Czaja et al. (2024) | Jones et al. (2021) | a) Humanoid (1) Leung et al. (2022) | a) Courses on the use of technical devices (2) Fields et al. (2020) Gadbois et al. (2022) | a) VR and hands-on activities (1) Fan et al. (2022) |
| b) Self-administered (2) Dang et al. (2023) Long (2023) | b) Educational and Psychological interventions (2) Esmaeilzadeh & Oz (2020) Nazari et al. (2021) | b) Courses on the use of technical devices (4) Mullins et al. (2020) Neil-Sztramko et al. (2020) Rolandi et al. (2020) Quinn (2021) |  | b) Asynchronous individual interventions (1) Dattilo et al. (2022) | b) Group interventions (3) Gusdal et al. (2023) Johansson-Pajala et al. (2023) Zarling et al. (2023) |  |  | b) Pets (3) Hudson et al. (2020) Tkatch et al. (2020) Lim (2023) | b) Befriending interventions (1) Adepoju et al. (2022) | b) Befriending interventions (1) Hsu et al. (2024) |
|  | c) Art and music interventions (6) Johnson et al. (2020) Galinha et al. (2021) Ho et al. (2021) Aydin & Kutlu (2021) Watson et al. (2023) Mouriño-Ruiz et al. (2024) | c) Group reminiscence therapy in combination with physical exercises (1) Ren et al. (2021) |  | c) Synchronous group interventions (5) Shapira et al. (2021a) Shapira et al. (2021b) Beauchet et al. (2022) Liu et al. (2023)  Yavuz & Şahin (2023) |  |  |  |  | c) Synchronous group interventions and exercises (1) Mierzwicki et al. (2023) | c) Health care services (2) Noh et al. (2021) Kousha et al. (2024) |
|  | d) Dance interventions (1) Hansen et al. (2021) |  |  | d) Synchronous exercises (Group or individual) (3) Weaver et al. (2022) Zengin Alpozgen et al. (2022) Hansen et al. (2024) |  |  |  |  | d) Virtual social centers (1) Palacios-Navarro et al. (2024) | d) Web-based interventions with subjective physical activities (1) Lippke et al. (2022) |
|  | e) Religious interventions (1) Borji & Tarjoman (2018) |  |  | e) Mixed (1) Funghi et al. (2024) |  |  |  |  |  | e) AI-based interventions with group meetings (1) Scherr et al. (2020) |
