# Supplementary material for: Interventions against loneliness and social isolation in older adults– a systematic review
Source: BMC Public Health. 2026 May 18;26:1562. doi: 10.1186/s12889-026-27683-9 (PMC13182138; doi:10.1186/s12889-026-27683-9)
Supplement: Supplementary file 8 [file 12889_2026_27683_MOESM7_ESM.docx]

Additional file 7 Table 5: Data extraction table

| Category | Title | Authors | Year of publication | Study aim | Study design | Total sample size (N) | Baseline characteristics (age, sex) | Intervention / control | Dose / Frequency / Duration | Measurement | Timepoints | Key conclusion |
| --- | --- | --- | --- | --- | --- | --- | --- | --- | --- | --- | --- | --- |
| Phone interventions | 21st Century Good Neighbor Program: An Easily Generalizable Program to Reduce Social Isolation in Older Adults | Sandu et al. | 2021 | Sharing an inexpensive longitudinal experiential service-learning program implemented by health professions and undergraduate student volunteers that aims to help alleviate loneliness in older adults while imparting meaningful experiences to volunteers. | Observational cohort study | 261 | Older adults aged 60+  162 (62%) female | Phone calls between student volunteers and older adults in the community. | Variable number of calls Scripts for the first three calls No information about frequency and duration of the intervention | Loneliness: UCLA Loneliness Scale (Version 3) | 2 measure points (Second call and first year follow-up) | No significant change in the UCLA loneliness score in the first year of follow up. Expansion of the social network of elderly individuals. |
| Web-based interventions - Group interventions | A codesigned web platform for reducing social isolation and loneliness in older people: a feasibility study | Johansson-Pajala et al. | 2023 | Evaluation of the feasibility of the Fik@ room regarding the outcomes of experienced loneliness and social networks, acceptability, resources, and recruitment of participants. | Feasibility study with a concurrent mixed methods design including quantitative and qualitative data | 30 | Older adults aged 65+ Mean age = 74.5 years (66-83); 24 (80%) female | Meetings in virtual Fik@ room with digital coffee tables and opportunities to start conversations in video, voice or chat. | Attending the Fik@ room at three specific times every week during the 12 week study period and additional possibility to use the room whenever they wanted | Loneliness: UCLA Loneliness Scale (Version 3)  Social networks: Social network questionnaire | 3 measure points (0 weeks, 6 weeks, 12 weeks) | The results showed that experienced loneliness decreased at the 6-week follow-up and increased at the 12-week follow-up but to a lesser extent compared with the loneliness experienced at baseline.  The results in the social network questionnaire showed that the overall frequency and satisfaction with social networks increased at the 6-week follow-up but decreased at the 12-week follow-up to an even lower level than at baseline. The Fik@ room is a feasible tool for older people to develop new friendships, reduce loneliness, and grow their social networks. |
| Community interventions - Art and music interventions | A Community Choir Intervention to Promote Well-Being Among Diverse Older Adults: Results From the Community of Voices Trial | Johnson et al. | 2020 | Testing the effects of the Community of Voices choir intervention on the health, well-being, and health care costs of racial and ethnically diverse older adults. | Multisite, cluster-randomized study | 390 IG: 208 CG: 182 | Older adults aged 60+  IG: Mean age = 71.8 years (59-93); 158 (76%) female  CG: Mean age = 70.5 years (59-93); 140 (77%) female | IG: Choir sessions including activities targeting three hypothesized pathways by which a choir could promote health and well-being: cognitive, physical, and psychosocial engagement.  CG: Wait-list (6 months) | 90-min choir sessions at the senior centers. Each choir met weekly for 44 weeks including 3-4 informal public performances. | Loneliness: NIH Toolbox for the Assessment of Neurological and Behavioral Function | Pre- and Post-test | There were significant group-by-time interaction effects for Loneliness (p = 0.02; ES = 0.34 and Interest in Life (p = 0.008, ES = 0.39). There was a decrease in Loneliness in the intervention group from baseline to 6 months, whereas the control group did not differentially change. Interest in Life increased in the intervention group but not the control group. |
| Videoconferencing interventions - Synchronous group interventions | A pilot randomized controlled trial of a group intervention via Zoom to relieve loneliness and depressive symptoms among older persons during the COVID-19 outbreak | Shapira et al. | 2021a | Exploring the efficacy of a short-term digital group intervention aimed at providing seniors with the tools and skills necessary for improving their coping ability during the COVID-19 outbreak. | Pilot randomized controlled study | 82  IG: 64  CG: 18 | Older adults aged 65+   IG: Mean age = 72.1 years; 52 (81%) female  CG: Mean age = 71.7 years; 14 (78%) female | IG: Online guided group sessions via Zoom consisted: a) a guided group discussion b) learning and practicing cognitive-behavioral techniques and skills such as relaxation, guided imagery of a ‘safe place’, identifying non-adaptive cognitive schemas, cognitive restructuring, and constructing positive self-talk.  CG: Wait-list (3.5 weeks) | Seven sessions lasting between 60 and 90 min Twice a week 3.5 weeks | Loneliness: 3-item UCLA Loneliness Scale | IG: 2 measure points (0 weeks, 3.5 weeks)  CG: 3 measure points (0 weeks, 3.5 weeks, 6 weeks) | Loneliness decreased within the intervention group from a mean (SD) of 5.4 (2.0) to 4.8 (1.7), which corresponded to a pre-post effect size of d = 0.32. Conversely, the wait-list control group loneliness score increased from 6.1 (1.9) to 6.4 (1.7), (pre-post effect size d = 0.166). Repeated measures mixed ANOVA revealed a significant main effect of time* group interaction (F(1,78) = 5.59, p = 0.02, η2 = 0.07, 95% CI [0.00, 0.16]). The significant interaction indicated that the groups differed in loneliness scores post-intervention. The main effect of time did not reach statistical significance (F(1,78) = 0.57, p = 0.45, η2 = 0.001, 95% CI [0, 0.06]). The calculated effect size for the between-group difference in loneliness scores was based on the mixed-model estimates and corre sponded to d = 0.58, indicating a medium effect size of the intervention. |
| Videoconferencing interventions - Synchronous exercises | A preliminary feasibility study of a virtual mentally stimulating activities program for older adults | Weaver et al. | 2022 | Examining preliminary feasibility of a virtually mentally stimulating activities (VMSA) program for community-dwelling older adults (aim 1) and the impact of VMSA on loneliness, depressive symptoms, and satisfaction with life (aim 2). | Pre-experimental case study | 4 | Older adults aged 60+  Mean age = 72.3 years (67-78); 4 (100%) female | The VMSA program started with a breathing exercise warm-up, followed by motor tasks (writing challenges with the nondominant hand) and challenging and novel cognitive tasks (describing favorite/least favorite smells, recalling childhood living spaces) and tactile item recognition. Various short cognitive exercises were designed to stimulate numerous cognitive domains (visual-spatial skills, executive function, language, crossing the midline for left/right hemisphere connections) and sensory stimulation within each session. | Eight sessions lasting 60 min Twice a week 4 weeks | Loneliness: 3-item UCLA Loneliness Scale | Pre- and Post-test | On the UCLA 3-Item Loneliness Scale, the average score across all participants at baseline (M = 5.0, SD = 1.4) was reduced by 1.5 points at post-test (M = 3.5, SD = 0.6; Figure 1). Individually, the scores of participants 1, 3, and 4 were indicative of “not lonely” at baseline and post-test. Participant 2’s 3-Item Loneliness Scale was in the clinical range of “lonely” at baseline but decreased to “not lonely” after the 4-week program. Results of the current study indicate efficacy, as evidenced in positive changes in loneliness and satisfaction with life from baseline to post-test within our sample. |
| Community interventions - Art and music interventions | A Randomized Waitlist-Controlled Trial of an Intergenerational Arts and Heritage-Based Intervention in Singapore: Project ARTISAN | Ho et al. | 2021 | Reporting the acceptability and effectiveness of the ARTISAN intervention in loneliness alleviation and enhancements in psychological well-being. | Open-label waitlist randomized controlled trial design (RCT) | 68  IG: 35  CG: 33 | Older adults aged 60+  IG: Mean age = 72.6 years (60-83); 29 (82%) female  CG: Mean age = 73.6 years (60-83); 27 (82%) female | IG: Group based intergenerational arts and heritage intervention with specific intervention components including guided museum tours, professionally led artmaking, guided storytelling and reflective writing that covered five intervention themes: (1) Discovering our National Heritage (2) Strengthening Social Bonds (3) Overcoming Adversities and Resilience (4) Building our Dreams and Aspirations (5) Sharing our Stories and Legacies  CG: Wait-list (5 weeks) | 15 hours 5 weeks | Loneliness: 8-item UCLA Loneliness Scale  Social connectedness: Social Connectedness Scale (SCS) | 3 measure points (0 weeks, 5 weeks, 10 weeks) | Within-group linear mixed model analyses further reveal that at 5-weeks follow-up, older adults in the intervention further experienced significant reduction in loneliness (1.97 vs. 1.75; 95% CI: −0.44 to −0.02, p = 0.034, d = 0.83) as compared to baseline. The rich qualitative data also provided strong support and insights to the intervention’s effects of building social connections, nationhood, and resilience. |
| Multicomponent interventions - AI-based interventions with subjective physical activities | Alexa, tell me more – about new best friends, the advantage of hands-free operation and life-long learning | Scherr et al. | 2020 | Exploring how to increase the participation of elderly people in social activities and how to reduce loneliness with the help of digital solutions. | Interventional study | 11 | Older adults aged 68+ Mean age = 76; 8 (73%) female | 1) Amazon Echo Show devices - Communicating with the device - Video call feature - Learning new things - Self-installed skills 2) Group meetings (coffee parties) | Variable Group meetings for 1,5 h after 1,5 months | Initial survey, intermediate survey, coffee parties, evaluation of devices (Open questions and scale questions) | Initial interview, Survey Intermediate interviews every 3 months | The social network of our participants seems to get closer to their household while the project progresses. The development shows that frequent loneliness decreased. People who, at the beginning, were lonely at least once per week were able to reduce this frequency to monthly or even more rarely. The outbreak of the Coronavirus in early spring 2020 and the fact that elderly people have to stay at home increased loneliness again for some, but for most participants, loneliness decreased even more as they were still able to have a video conversation with other people. Most of the participants also said that having such a device is beneficial for their health because they are able to do more things independently from home compared to the time when they had no Alexa. |
| Videoconferencing interventions - Synchronous group interventions | Benefits of a 3-month cycle of weekly virtual museum tours in community dwelling older adults: Results of a randomized controlled trial | Beauchet et al. | 2022 | Examining whether a 3-month cycle of weekly virtual tours of the Montreal Museum of Fine Arts (MMFA) may have improved feelings of social inclusion, wellbeing and quality of life, and reduced physical frailty in older adults living within the community of Montreal. | Randomized controlled study | 106  IG: 53  CG: 53 | Older adults aged 65+   IG: Mean age = 75.0 years; 38 (72%) female  CG: Mean age = 74.3 years; 48 (91%) female | IG: Participation in a virtual MMFA (Montreal Museum of Fine Arts) guided tour via Zoom seperated into three phases: (1) Presentation of the visit objective (2) A dialogic-style tour with trained museum guides (3) An open-ended discussion after the tour The tour content consisted of a combination of images of artworks, live discussions animated by the tour guides, ancillary information on the artworks or artists from tour guides, and pre-recorded videos about specific works or artists.  CG: Participants were asked to avoid participation in any arts-based activity 3 months ahead of the study and over the 3-month period of the study itself. | 45-minute virtual guided tours + 15-minute informal discussion Once a week  3 months | Social isolation: DSSI (11-item Duke Social Support Index) | Pre- and Post-test | There were significant greater mean scores for DSSI (P ≤ 0.001) at M3 compared to M0 in the intervention group. No significant change in all scales’ scores between M0 and M3 was found in the control group. Inter-group comparisons showed that DSSI mean scores were significantly higher in the intervention group compared to the control group at M3 (P < 0.033), while significant difference was found at M0. Participation in weekly virtual MMFA tours was significantly associated with improvements in all scales (P ≤ 0.012). The findings of this RCT show that the 3-month cycle of weekly virtual MMFA tours had multidimensional benefits in participating older adults. Social isolation decreased and both physical and mental health improved significantly. |
| Individual interventions - Trainer-administered | Benefits of a psychosocial intervention programme using volunteers for the prevention of loneliness among older women living alone in Spain | Lorente-Martínez et al. | 2021 | Analyzing the effectiveness of a psychosocial intervention programme in preventing loneliness and increasing self-efficacy and social participation among older women living alone in Spain. | Non-randomized quasi-experimental study | 48  IG: 34  CG: 14 | Older women aged 65+  IG: Mean age = 77.62 years; 34 (100%) female  CG: Mean age = 77.36 years; 14 (100%) female | IG: The Acompaña-Té programme is a psychosocial support intervention based on the cognitive model of loneliness (Peplau & Perlman, 1979).  Three components: 1. Conversation (all sessions): a multi-purpose social component used to establish a good interpersonal relationship between the volunteer and the older person. 2. Attribution retraining (sessions 2 to 4): a cognitive component for enhancing perceived self-efficacy in situations of loneliness.  3. Behavioural activation (sessions 4 to 9): a behavioural component for devising a social activity plan that promotes social participation in the local area and enhances feelings of social integration.  CG: Wait-list (6 months) | 9 sessions of 2.5 hours (25 hours) Once a week Two months | Loneliness: UCLA Loneliness Scale (Version 3)  Social participation: SSPI (Subjective Social Participation Index) | IG: 3 measure points (0 months, 2 months, 6 months)  CG: 2 measure points (0 months, 2 months) | Women in the EG perceived their social participation to be higher after the intervention, but the increase in their mean score was not significant when the hypothesis was tested. Women in the CG perceived that their social participation was lower in the post-test, although changes in social participation scores were not statistically significant. The pre-intervention mean scores indicated an absence of clinical loneliness in both the EG and CG. In the post-test assessment, a slight increase in the scores of both groups was observed, indicating a reduction in the level of loneliness, but these changes were not statistically significant. |
| Application-based interventions | Breaking Social Isolation for Older People Living Alone with Technology | Dinet et al. | 2023 | Presenting two studies investigating attitudes of older people towards different technologies to break their social isolation and investigating the impacts of Immersive Virtual Reality (IVR) on physical and mental dimensions. The first study, based on focus groups conducted with 23 older people (aged from 65 to 93 years-old), is aiming to collect attitudes and opinions about different technologies to help them to break social isolation. The second study, based on an experiment conducted with 42 older people (aged from 63 to 85 years-old), is aiming to investigate the impact of Immersive Virtual Reality (IVR) on physical and mental dimensions. | Experimental study | 42  IG: 21  CG: 21 | Older adults aged 63+  IG: Mean age = 78.4 years; 13 (61%) female  CG: Mean age = 77.8 years; 14 (67%) female | IG: The IVR (Immersive Virtual Reality) used in our study was specifically created for a series of experiments investigating the behaviors of pedestrians with specific needs (older adults, adults with motor impairment) in urban areas. This IVR has been developed by Human Games© company and requires the use of an HTC Vive headset. In this IVR system, pedestrian / user can freely navigate in a city (crossing streets, moving, stopping to move). Other pedestrians and vehicles are also present.  CG: Standard-of-care | 15 minutes per day Daily 4 months | Loneliness: (Revised UCLA) loneliness scale | Pre- and Post-test | The loneliness score (perception of social isolation) decreases significantly for participants using the IVR system (𝑝. < 0.001), while this score remains stable for the control group. |
| Phone interventions | Combating Loneliness in Older Adults during the COVID-19 Pandemic: Findings from a Volunteer-Based Program in Greece | Balta et al. | 2023 | This study aims to investigate any association between COVID-19 cases in Greece and the number of Loneliness Helpline calls at the Friendship at Every Age program and to assess whether the interconnection part of the program, which interconnects older adults with volunteers, can combat loneliness/social isolation in older adults. | Non-randomized noncontrolled intervention study | 168 | Older adults aged 60+  121 (72%) female | During the first three years of implementation, the program has specifically supported older adults and consists of two main pillars of action:  (a) The Loneliness Helpline: Calls with volunteers to keep company to lonely older adults and to offer human contact (b) The interconnection part (IC): Regular telephone communication between the beneficiaries (older people) and their assigned trained volunteers to create new friendships. | At least 30 minutes At least once per week July 2020 - January 2023 | Loneliness: UCLA Loneliness Scale | Pre- and Post-test | A significant decrease in the UCLA-LS was observed at follow-up, compared with baseline UCLA-LS (difference = −0.85 (2.0) units, p < 0.001). Similar results were found when beneficiaries were categorized as “lonely” and “not lonely” (based on the UCLA Loneliness Scale score). Specifically, feeling “not lonely” was reported by 27.9% of the beneficiaries at follow-up, compared to 14.6% at baseline (p < 0.001). Loneliness Helpline calls increased during COVID-19 outbreaks, while the interconnection part had a positive impact on older adults, reducing their feeling of loneliness. |
| Community interventions - Dance interventions | Dance Intervention Affects Social Connections and Body Appreciation Among Older Adults in the Long Term Despite COVID-19 Social Isolation: A Mixed Methods Pilot Study | Hansen et al. | 2021 | Testing the effects of an intervention designed to facilitate embodied social connections among older adults in the long term. | Mixed methods pilot study | 10 | Older adults aged 60+  Phase I: Mean age = 67.5 years; 7 (70%) female  Phase II: Mean age = 69.11 years; 8 (80%) female | Free community dance sessions. Each day started with a 10–15 min circle greeting followed by a 20 min warmup. A 45–60 min creation session was facilitated on each side of the mid-session break and every day ended with a circle debrief, inviting participants to share impressions and experiences from the day. | 3,5 hours 4 days | Loneliness: UCLA Loneliness Scale (3I-UCLA) | 4 measure points (Phase I: 0 days, 4 days, Phase II: 4 months, 5 months) | Taking into account all four timepoints, a significant main effect on loneliness was found [F(3,27) = 4.52, p = 0.011, ηp2 = 0.33, see Figure 2]. At the Phase II time point we found a statistically significant increase in overall loneliness across the full sample of participants from scores reported post-intervention [t(9) = −2.54, p = 0.032, d = 0.80]. Note that the ability to sustain or improve body appreciation through COVID-19 measures did not eliminate the increased experience of loneliness. We do not know whether individuals in this demographic would have felt even more lonely without the positive effect of the intervention, but it seems that it is possible to have increasingly positive feelings about the body while also feeling lonely due to isolation or distancing from social connections. |
| Web-based interventions | Development and evaluation of the information and communication technology-based Loneliness Alleviation Program for communitydwelling older adults: A pilot study and randomized controlled trial | Ae-Ri et al. | 2023 | Developing an integrated ICT-based Loneliness Alleviation Program (LAP), including physical, psychological, and interpersonal support components, for older adults living in the community and assess its efficacy. | Pilot study and randomized controlled study | 40  IG: 20  CG: 20 | Older adults aged 65+  IG: Mean age = 76.50 years; 16 (80%) female  CG: Mean age = 76.50 years; 18 (90%) female | IG: YouTube videos delivered through cell phone text messages and KakaoTalk messenger 1st session: Face-to-face introductions and instructions on how to watch the YouTube videos and a booklet containing program explanations and workbooks 2nd to 12th session: YouTube videos, contents consisted of smartphone usage, horticultural therapy, drug management, laughter therapy, music exercise therapy, and sleep relaxation therapy  CG: Mushroom growing kit and activity sheets | 12 sessions Twice a week 6 weeks | Loneliness: Revised UCLA Loneliness Scale | Pre- and Post-test | Loneliness scores in the experimental group was 39.6 +- 12.2 points at pre-intervention and 31.0 +- 7.5 points at post-intervention, indicating a statistically significantly decrease in the scores after the intervention (F = 5.340, p = 0.001). |
| Individual interventions - Trainer-administered | Effect of integrated approach of yoga therapy on loneliness in elderly: An interventional study | Panigrahi et al. | 2023 | Knowing the effect of integrated approach of yoga therapy on loneliness of elderly people. | Interventional study | 50 | Older adults aged 60+  (60-80), 20 (40%) female | After Morning Prayer for 2 minutes the practice session started with the following yogic module: 1. 10-minute lectures about IAYT, food, emotion culture, concepts, and how to deal with loneliness according to yogic principles.  2. Loosening Exercise (15 minutes)  3. Jogging-5 minutes Tadasana (5 rounds), Tiryak Tadasana (5 Rounds), Kati chakrasana (5 rounds)-10 minutes  4. Surya namaskar-6 rounds-10 minutes  5. Savasna-4 minutes  6. Yogic Breathing in Savasana-3 minutes  7. Anulom Vilom Pranayama-5 minutes  8. Om chanting-3 minutes  9. Cyclic meditation (25 minutes)  10. Participating in Mandir Arati  11. Evening Satsanga twice in a week  12. Cleansing the mandir premises and surrounding on Sunday | 90 minutes 6 days a week 12 weeks | Loneliness: UCLA Loneliness Scale | Pre- and Post-test | The results showed that the program was effective for reducing loneliness and depression and for increasing the laughter index of older adults. |
| Community interventions - Educational and Psychological interventions | Effect of Psychosocial Care Model Applied in an “Elderly Day Care Center” on Loneliness, Depression, Quality of Life, and Elderly Attitude | Esmaeilzadeh & Oz | 2020 | Evaluating the effect of the model applied in order to meet psychosocial care needs of elderly people in an elderly day care center on loneliness, depression, quality of life, and elderly attitude. | Interventional study | 39 | Older adults aged 65+  34 (87%) female | Psychosocial care model with visual methods, question answer and discussion technique, homework, and warming games Topics covered in sessions: 1. Introduction of the program, creation of group consciousness, adaptation to elderliness 2. Creating self-awareness and consciousness 3. Increasing self-esteem 4. / 5. Problems and solutions in old age 6. Coping with regret in the past and uncertainty and death 7. Giving meaning to the life, effective coping and adaption to elderliness 8. Improving the quality of life 9. Completion | 9 sessions of 2 h Once a week 4 months | Loneliness: UCLA Loneliness Scale (turkish version) | Pre- and Post-test | Comparison of points that the individuals obtained from prior to and after intervention is shown. In the paired ttest, the elderly individuals obtained x– = 47.38 ±8.64 point prior to UCLA LS, x– = 39.62 ± 8.67 point after the intervention. The difference between these scores was statistically significant (P < 0.05). It was found that there was a decrease in the scores of loneliness after the intervention compared to the prior to intervention, in other words, the feeling of loneliness after the intervention was less experienced. |
| Multicomponent interventions - Health care services | Effectiveness of a community-based integrated service model for older adults living alone: A nonrandomized prospective study | Noh et al. | 2021 | Applying the CBIS model among older adults living alone and evaluating its effectiveness. | Prospective non-randomized study | 877  IG: 331  CG: 546 | Older adults aged 65+  IG: 261 (79%) female  CG: 430 (79%) female | IG: CBIS model The eight healthcare services are:  (1) community primary medical system linkage  (2) medical/health product linkage support (3) accompaniment to the hospital (4) emergency support (5) emotional support (contact via phone call or visit, emotional support program, counseling)  (6) cognitive enhancement program (playing cognitive function enhancing games) (7) self-management program for chronic illness (8) supervised medication administration.  The five social care services are:  (1) meal support (visiting meal support, meals on wheels, or cooking class) (2) cleaning/washing (3) delivery of ingredients (4) improving the residential environment (5) community outings. CG: Standard-of-care | 10 min to two hours 6 months | Loneliness: UCLA Loneliness Scale  Social support: ENRICHD ESSI | Pre- and Post-test | Loneliness scale significantly reduced from 43.097 to 38.830 in the intervention group, as compared to the control group in which it decreased from to 41.973 to 39.603.  The least-square means of the ESSI were not significant. |
| Mixed analogue interventions - Trainer-administered | Effectiveness of a multicomponent intervention to reduce social isolation and loneliness in community-dwelling elders: A randomized clinical trial | Hernández-Ascanio et al. | 2021 | Assessing the effect of a multicomponent intervention on reducing social isolation and loneliness and improving the quality of life in community-dwelling older adults. | Cluster-randomized controlled clinical trial | 119  IG: 63  CG: 56 | Older adults aged 65+  Mean age = 81.79 years; 91 (77%) female | IG: Adaptation of the CARELINK programme Three components: (1) Communication and social contact  • Conversation while performing daily activities • Discussion about news (2) Feelings of competence and self-control • Evocation and reminiscence of motivating activities or desirable participation contexts.  • Identification of causes, factors related to the loneliness and isolation situation.  • Planning of activities that the subject is able to perform.  • Training in control and coping skills.  • Positive rein (3) Participation in social activities • Information on social community resources favouring participation and connection with other people.  CG: Standard-of-care | 6 home sessions of at least 30 min 5 telephone calls of at least 20 min 4 months | Loneliness: The Jong-Giervald Loneliness scale  Social isolation: Duke-UNC Functional Social Support Questionnaire (DUFSS) | IG: 3 measure points (0 months, 4 months, 6 months)  CG: 2 measure points (0 months, 6 months) | Regarding the effect of the intervention, no statistically significant difference in the total score of the social isolation variable was found between both groups at the end of follow-up. However, regarding the specific dimension of confidential support, we found a very close level of statistical significance (p = 0.058) according to the intentionto-treat analysis.  When analysing the data disaggregated by group, in the experimental one, significant differences were found between the results obtained at baseline (T1) and at the end of the study (T2) in all dimensions. No differences were found in the control group in this regard. The total DUFFS (social isolation) scores in the experimental group improved by almost three expected points (scores ranged from 25.00 to 27.94, p = 0.005) between the T1 and T2. In addition, the effect of the intervention on this variable was maintained and still improved 2 months after the end of the intervention, that is, at T3 (reaching 28.5, p = 0.002 vs. T1). Concerning the comparison of means associated with the loneliness variable, no statistically significant differences were found between both groups for any of the scores when analysing them at T1 and at T2. However, if we compare paired means in the experimental group, an improvement in emotional loneliness scores between T2 and T3 was found (Wilcoxon test; p = 0.012). |
| Multi-component interventions - Health care services | Effectiveness of an Informal Home Care Support Intervention Program to Reduce Loneliness and Improve Quality of Life among Lonely Community-Dwelling Older Adults: A Feasibility Study | Kousha et al. | 2024 | Evaluating the effectiveness of a peer-to-peer informal home care support intervention program (HoSIP) in reducing loneliness and improving quality of life among lonely community-dwelling older adults, as a feasibility study. | Quasi-experimental pre-post study | 87 IG: 36 CG: 51 | Older adults aged 60+ IG: Mean age = 68.36, 83.3% female CG: 90.2% female | IG:  - Online social group (HAMDAM) for daily needs - Educational and art classes - Emotional support - Instrumental support - Birthday parties  CG: No intervention | Daily 12 weeks | Loneliness: 20-item UCLA Loneliness Scale (Version 3)  Social network: Six-item Lubben Social Network Scale  Social support: 12-item Multidimensional Scale of Perceived Social Support | 4 measure points (Pre-intervention, post-intervention, 3 months, 6 months) | The results of the RMANOVA for loneliness, quality of life, and perceived social support indicated that the assumption of equality of means in different stages of measurement was rejected: (P<0.001, F=15.915), (P<0.001, F=16.499), and (P<0.001, F=8.362), respectively; there was a significant relationship between different stages of measurement. The between-group test results indicated significant interaction effects within the intervention and control groups: (P=0.006, F=7.872), (P<0.001, F=26.893), and (P<0.001, F=16.774), respectively. We found a significant relationship between the outcome variables, including feelings of loneliness (P<0.001) and quality of life (P<0.001), at different stages of measurement.  Utilizing the existing capabilities of older adults to provide online and face-to-face care services can be a cost-effective way to improve their quality of life and reduce loneliness. |
| Mixed analogue interventions - Trainer-administered | Effectiveness of INOSEL program consisting of Integrative Nursing and Omaha System for older women feeling loneliness: a randomised controlled trial | Ilgaz & Gözüm | 2023 | Evaluating the effect of a program based on IN principles and the OS on the physical, mental, social and spiritual health of community-dwelling older women experiencing a high level of loneliness. | Randomized controlled study | 69  IG: 36  CG: 33 | Older women aged 65+  69 (100%) female | IG: (1) Group-based intervention: In this part of the intervention, walking, picnics, sightseeing, cinema, theatre and group training sessions on healthy nutrition, stress management and interpersonal relations were held for the participants. (2) Person-centered intervention:  Each participant’s health problems were determined according to the OS Problem Classification Scheme. The most common health problems identified were social interaction, interpersonal relationships, physical activity, pain, circulatory and intestinal function, sleep and rest patterns and nutrition. Interventions were chosen from the OS Nursing Intervention Scheme according to the individual problem defined by the researchers in the Problem Classification Scheme.  CG: Not taking part in any intervention, except for the services provided in the community. | 12 weeks | Loneliness: Loneliness scale for the elderly  Social support: Multidimensional Scale of Social Support   Social insclusion: Social Inclusion Scale | Pre- and Post-test | According to ITT, INOSEL and control groups showed an improvement in loneliness mean score after 12 weeks of the INOSEL program. In both groups, the loneliness levels of older women who “felt significant loneliness and felt very intense loneliness” decreased to the level of “acceptable loneliness and not feeling loneliness”. However, the decrease in loneliness mean score and loneliness level was higher in the INOSEL group as compared to control group. The older women’s level of physical activity and health status, social support, social inclusion, well-being and spirituality perception scores increased significantly in the INOSEL group (p < 0.001). In comparison, the control group showed no change (p > 0.05). |
| Robot interventions - Pets | Effects of a cognitive-based intervention program using social robot PIO on cognitive function, depression, loneliness, and quality of life of older adults living alone | Lim | 2023 | Improving the cognitive function of older adults living alone by using the social robot PIO and confirming the effect of reducing depression and loneliness and improving QoL through the interaction of the social robot PIO. | Non-equivalent control group pre-test–posttest design | 64  IG: 31  CG: 33 | Older adults aged 65+  IG: 65-89 years; 29 (94%) female  CG: 65-89 years; 28 (84%) female | IG: The program was structured by a storytelling of the process behind the parrot-shaped robot PIO hatching from an egg and growing into an adult robot. Each session started with the themes of “meeting with the social robot PIO” and “recalling the previous program.” In addition, gymnastics was performed with the social robot PIO to relieve tension and improve intimacy with the robot. Before the 6th session, “Teaching movements and gymnastics,” gymnastics was performed while watching a video, and after the 6th session, gymnastics was performed while watching the movements of the social robot PIO. The ending involved “Expressing your impression about the program” and “Saying goodbye to social robot PIO”  CG: Standard-of-care | 12 sessions of 50 min Twice a week 6 weeks | Loneliness: Revised UCLA Loneliness Scale (RULS) | Pre- and Post-test | For the loneliness variable, the difference between the prepost scores of the experimental and control groups was statistically significant (t = −4.27, p < 0.001). The experimental group decreased by −11.26 ± 9.34, and the median (IQR) decreased to −12 (14), the control group decreased by −0.55 ± 10.64, and the median (IQR) was 1 (16). The results showed that the cognitive function scores improved, depression and loneliness scores decreased, and the difference between groups in the pre-post change was statistically significant. |
| Mixed technological interventions - Virtual social centers | Effects of a home care community-dwelling intervention on cognition, mental health, loneliness and quality of life in elder people: The VERA study | Palacios-Navarro et al. | 2024 | Validating a technological system based on videoconferences that allows users to receive comprehensive attention focused on the different biopsychosocial spheres. | Quantitative quasi-experimental study with a longitudinal pretest and posttest within subject design. | 7 | Older adults aged 60+  Mean age = 71 years (61-92); 5 (71%) female | Multimodal approach based on a videocall system including sessions with social workers, psychologists, sociocultural entertainers and physiotherapists. VERA is a virtualsocial center whose objective is to transfer,with the help of technology,the entire activity of any traditional social center to the users’ homes, in order to eliminate physical and distance barriers that prevent movement. The platform makes services available to users that range from psychological care and social workers to facilitating the individual and collective integration of the elderly or promoting their maximum degree of autonomy. The system consists of a TV set, remotecontrol, video camera and, if necessary, a router. | Weekly 6 months | Loneliness: ESTE scale (ESTE-R) | 3 measure points (pre-test, intermediate, post-test) | The implemented program has managed to significantly reduce (and with a high effect size) perceived loneliness in the first part of the intervention, remaining without significant changes until the end of the intervention. |
| Mixed analogue interventions - Trainer administered | Effects of social prescribing pilot project for the elderly in rural area of South Korea during COVID-19 pandemic | Kim et al. | 2021 | Identifying the effectiveness of the social prescribing model in the COVID-19 pandemic era by investigating its impact on depression, loneliness, and attitudes toward social participation, self-efficacy, and self-esteem in elderly people in South Korea. | Pilot project | 10 | Older adults aged 65+  Mean age = 82.0 years; 10 (100%) female | IMusic storytelling, Self-help group, Gardening, COVID-19 prevention | Sessions of 30-90 min Once a week 10 weeks | Loneliness: UCLA Loneliness Scale  Social participation attitude: Social participation scale for the elderly living alone | Pre- and Post-test | Mean scores for the attitude toward social participation were 29.90 (SD 5.7) and 35.10 (SD 3.3) before and after intervention, respectively. In the progress evaluation phase, subjects' characteristics were evaluated. According to the findings, the mean loneliness score was 25.60 (SD 2.5) before intervention, and 22.20 (SD 5.0) after. First, loneliness tended to decrease statistically (Z = -1.684, P < .1). Furthermore, the attitude toward social participation statistically and significantly increased (Z = -2.173, P < .05) after intervention. For the subjects who participated in all three social prescribing intervention programs (music storytelling, the self-help group, and gardening), scores for both depression and loneliness decreased statistically and significantly, whereas social participation attitude and self-esteem statistically and significantly increased. |
| Videoconfoerencing interventions - Asynchronous individual interventions | Effects of Video-Assisted Leisure Education on Leisure, Loneliness, and Affect of Older Adults | Dattilo et al. | 2022 | Examining effects of the leisure education program, Project BHEL. | Nonrandom pretest, post-test control group experimental design | 52  IG: 20  CG: 32 | Older adults aged 70+  IG: Mean age = 85.5 years (71-98); 16 (80%) female  CG: Mean age = 84.6 years (72-96); 25 (78%) female | IG: Project BHEL with instructional videos, discussion, and learning activities to promote goal-setting and problem-solving strategies • Initial pre-session video (9 min, 29 sec): Study introduction, survey guidelines, directions for the leisure day diary, discussion guidelines and roles • First video (15 min, 11 sec): Leisure and ways free time use tends to change as we age, overview of upcoming four videos identifying benefits targeted in each session (social, physical, educational, psychological) • Second video (16 min, 43 sec): Review and leisure involving social contact, making a plan to reach a social goal • Next three videos: Individual goals and a plan to do recreation activities that result in physical fitness, learning and being happy • Seventh video (7 min, 8 sec): Problem-solving strategy for their final goal on engaging in leisure experience and summary and conclusion of the entire program  CG: Standard-of-care | Sessions of 1 h 7 sessions | Loneliness: UCLA Loneliness Scale | Pre- and Post-test | There were no significant effects on loneliness for the treatment group (F[1,41] ¼ 2.15, p¼.15, ES ¼ 0.41), time (F[1,41] ¼ 2.17, p¼.15, ES ¼ 0.41), nor their interaction (F[1,41] ¼ 1.71, p¼.20, ES ¼ 0.37). Although results did not reveal a significant decrease in loneliness post intervention, the occurrence of loneliness scores for the treatment group in this study trending toward a decrease and remaining unchanged in the control group is worth considering. |
| Community interventions - Group meetings | Efficacy of group reminiscence therapy based on Chinese traditional festival activities (CTFA-GRT) on loneliness and perceived stress of rural older adults living alone in China: a randomized controlled trial. | Li et al. | 2022 | Exploring the efficacy of group reminiscence therapy based on Chinese traditional festival activities (CTFA-GRT) to mediate the loneliness and perceived stress of older adults living alone in rural China. | Randomized controlled study | 60  IG: 30  CG: 30 | Older adults aged 60+  IG: Mean age = 65.20 years; 18 (60%) female   CG: Mean age = 65.56 years; 20 (67%) female | IG: Group-reminscence therapy based on Chinese traditional festival activities (CFTA-GRT) • Month • Festivals • Theme • Objectives • Acitvity content • Homework  CG: Wait-list | 8 sessions for four hours Once per month 8 months | Loneliness: UCLA Loneliness Scale | 3 measure points (0 months, 8 months, 11 months) | The CTFA-GRT yielded a benefit for the perceived stress and loneliness of rural older adults living alone in the intervention group. Combined with a simple effects test, the PSS and UCLA-LS of those in the intervention groups significantly decreased at eight months after the baseline (F PSS = 23.540, F UCLA-LS = 40.159; all P < 0.01). In addition, the sustainable effect of this program lasted three months after the intervention (F PSS = 65.756, F UCLA-LS = 60.983; all P < 0.01). |
| Phone interventions | Evaluating Feasibility, Value and Characteristics of an Intergenerational Friendly Telephone Visit Program During the Covid-19 Pandemic | Kumar et al. | 2023 | Evaluating Feasibility, Value and Characteristics of an Intergenerational Friendly Telephone Visit Program During the Covid-19 Pandemic. | Single-arm interventional study | 10 | Older adults aged 65+  Mean age = 73.43 years (70-84); 9 (90%) female | The Harvard Concordium is an undergraduate-run volunteer program which connects students with community-dwelling older adults experiencing loneliness. Students are paired with older adults based on common interests. They are expected to interact at least once per week and to continue the relationship for at least one year. | 8 sessions for 30 minutes Weekly One year | Loneliness: PROMIS Social Isolation Scale | Pre- and Post-test | For the seven older adults who completed the study, median pre- to post study change scores for loneliness, behavioral activation and positive affect and well-being all lie in a positive (improved) range whereas the median change score for perceived stress lies close to 0 (no change). |
| Community interventions - Art and music interventions | Evaluation of effectiveness of an art-based museum intervention in reducing loneliness among older adults (ArtGran): a quasi-experimental study | Mouriño-Ruiz et al. | 2024 | Assessing the effectiveness of ArtGran in reducing loneliness and its negative effects on health in community-dwelling older adults in 2022 in Barcelona. | Quasi-experimental study | 138 IG: 63 CG: 75 | Older adults aged 70+  IG: 53 (84.1%) female  CG: 65 (86.7%) female | IG: Meetings in the museums The sessions were based on Visual Thinking Strategies, an educational approach used in museums, which utilises art to teach communicative, thinking, and visual competency skills through art observation. Sessions were organised into 4 main parts:  1) Participant welcome and introduction to the working topic;  2) Artwork observation and topic development in the exhibition spaces;  3) Engagement with the topic through a related activity;  4) Collective discussion and session closure  CG: No intervention | 10 sessions of 2 h  Weekly 2 months | Loneliness: Este II Social Loneliness Scale | Pre-intervention and post-intervention | No significant differences in pre-post changes were observed between groups in self-perceived health or quality of life. However, at the end of the intervention, the number of individuals with a low level of loneliness increased by 4.20% among the participants with high attendance (6–10 sessions), which means that they have improved as they have moved from higher levels of loneliness to the low level. In the CG, contrarily, it decreased by 19.60% (P = 0.014).  Participants who attended more than half of the sessions were 36% more likely than the participants in the CG to have a low level of loneliness (aPR [95%CI = 1.36 [1.07–1.73]).  Compared with the CG, participants in the IG attending more than half of the sessions had lower levels of loneliness (aPR = 1.36; 95%CI: 1.07-1.73). |
| Robot interventions - Humanoid | Exploring the Presence of Humanoid Social Robots at Home and Capturing Human-Robot Interactions with Older Adults: Experiences from Four Case Studies | Leung et al. | 2022 | Understanding the experiences and perspectives of older adults and their family caregivers on using a humanoid social robot over a two week period at home and to explore the potential benefits and barriers of human-robot interactions at home. | Case studies | 4 | Older adults aged 65+  3 (75%) female | Interaction with a humanoid social robot called KaKa This robot is designed in a way that it simulates human-robot interactions with a human voice from the robot responding to a human’s verbal instructions or initiating conversations by the robot to a human at a specific time. This cultivates an environment for human-robot interactions and human-human interactions via the robot. Four features were selected based on the findings of the literature review as well as the comments from the potential users of the robot:  (1) interactive modalities (2) calendar planning and task reminder (3) promoting healthy lifestyle  (4) puzzle games | Daily 2 weeks | Semistructured interviews | Post-intervention | The utilization of Ka Ka was reported to reduce feelings of loneliness and boredom among older adults living alone. |
| Web-based interventions - Individual interventions | Feasibility, acceptability, and preliminary efficacy of an internet-based CBT intervention for loneliness in older adults: A pilot RCT | Dworschak et al. | 2024 | Investigating the feasibility, acceptability, and preliminary efficacy of a newly developed internet-based cognitive behavioral therapy intervention for loneliness in older individuals using a pilot ran domized controlled trial. | Pilot randomized controlled study | 36  IG: 18  CG: 18 | Older adults aged 65+  IG: Mean age = 70.83 years (65-87); 13 (72%) female  CG: Mean age = 72.06 years (65-87); 14 (78%) female | IG: Internet-based program "NümEinsam" consisting seven modules focusing on different topics. The main focus of the intervention was on identifying and adapting maladaptive thoughts and cognitions associated with loneliness. In line with this, the program provided both a theoretical model that was sought to help participants identify and understand their beliefs and behaviors, as well as strategiesand behavioral experiments to challenge and question them. CG: Wait-list | 7 modules One module per week 7 weeks | Loneliness: UCLA Loneliness Scale | Pre- and Post-test | Although a small between-group effect was observed on the UCLA favoring the treatment group over control condition, the main model did not reveal a significant group by time interaction. The addition of control variables to the model did not result in any significant changes. In terms of the primary outcome loneliness, we did not find a significant difference between groups; however, results indicated a trend towards lower reported loneliness levels at post-assessment in the treatment compared to the control condition. |
| Mixed technological interventions - Courses on the use of technical devices | Findings From Talking Tech: A  Technology Training Pilot Intervention to Reduce Loneliness and Social Isolation Among Homebound Older Adults | Gadbois et al. | 2022 | Reporting findings from a novel pilot intervention, called Talking Tech, designed to reduce loneliness and social isolation in homebound older adults by providing one-on-one, in-home technology training, a tablet, and the internet, to promote digital literacy and participation in a virtual senior center. | Qualitative interviews and quantitative pre- and post-intervention surveys | 21 | Older adults aged 60+  Mean age = 73 years (61-93); 13 (62%) female | Talking Tech intervention This training and support focused on how to use a tablet, access the internet, and participate in a virtual senior center program, called Well Connected. Well Connected is a national phone- and internet-based program, which provides over 70 sessions per week on topics including virtual travel, support and conversation groups, bingo, and language learning, among others. Participants were paired with a TechMate and given a tablet and a hotspot connection for 1  year. | 14 weeks Hotspot connection provided for one year | Loneliness: Qualitative interviews, Three-Item Loneliness Scale | Pre- and Post-test | Baseline, preintervention survey data were compared with postintervention, follow-up data. Across participants, there were no significant differences for any of the measures between baseline and follow-up. Loneliness scores were, however, trending in right direction, with a mean score of 6.00 at baseline reducing to 5.44 on the follow-up survey. Participants and TechMates reported a number of benefits participants received from Talking Tech, including several ways that Talking Tech helped reduce loneliness and social isolation. Pilot participants report benefits including greater technological literacy and use, social connections to existing and new networks, and use of a virtual senior center, despite a lack of significant quantitative findings on the impact of the intervention on loneliness. |
| Individual interventions - Trainer-administered | Holistic Wellness Coaching for Older Adults: Preliminary Evidence for a Novel Wellness Intervention in Senior Living Communities | Fullen et al. | 2022 | Examining the feasibility and effectiveness of the wellness coaching program within senior living communities. | Pilot study | 79 | Older adults aged 70+  Mean age = 84.62 years (71-97); 53 (67%) female | Wellness coaching program Multidimensional approach to wellness - Efforts to care for one's body - Prresent engagement in supportive relationships - Self-efficacy and a sense of control over one's life - Ability to cope with challenging emotions - Pursuing meaning and purpose - Living out a sense of purpose - Developing constructive views of aging and successfully navigating life transitions - Enviromental wellness Self-determintation theory (SDT) | 20-30 min  Daily 9 weeks | Loneliness: Three-Item Loneliness Scale | 3 measure points (pre-program, post-program, 1-month follow-up | Compared to the pre-program, there was a significant decrease in loneliness post-program (p = 0,001) and at the follow-up (p = 0.035). Repeated measures ANOVAs revealed positive changes in resident loneliness and relatedness. Some of the results persisted at follow-up. |
| Mixed analogue interventions - Courses on the use of technical devices | Impact of the Age-on tablet training program on social isolation, loneliness, and attitudes toward technology in older adults: Single-group pre-post study | Neil-Sztramko et al. | 2020 | Exploring whether taking part in a real world offering of a 6-week tablet training program for older adults reduced social isolation or loneliness or improved quality of life. | Single-Group Pre-Post Study | 32  IG: 21  CG: 11 | Older adults aged 60+  Mean age = 76.3 years; 20 (63%) female | IG: Series of workshops covering the basic features of a tablet. The first week of classes focused on learning the basic features of an iPad (eg, powering on and off, volume, locating controls) and locating the variety of available apps. In subsequent weeks, participants learned how to use specific applications, including using the internet, taking and viewing photographs, sending and receiving emails, and using basic apps (eg, the Maps app, the Clock app, and Siri). The modules were accompanied by a participant workbook that included session content, homework, and additional information to help the participants learn the material.  CG: Care as usual | Sessions of 2 hours Weekly 6 weeks | Loneliness: De Jong Giervald Loneliness Scale  Social isolation: Duke Social Support Index (DSSI)  Social network:12-item Lubben Social Network Scale | 3 measure points (pre-program, post-program, follow-up after 1 month) | There were no significant differences in social isolation when measured either as a total score or as interaction or support sub-scores between baseline and end of study or at 1-month follow-up (Table 2). Moreover, no differences were found for loneliness, quality of life, or social support at 1-month follow-up. After completing the program, no differences in social isolation, loneliness, social support, or quality of life were found. Frequency of tablet use increased and the attitudes of the participants toward technology improved. |
| Application-based interventions | Improving the Social Connectedness of Older Adults Through Digital Social Gaming - A Pilot Study | Janssen et al. | 2023 | Exploring the possibility of this game to activate and engage older adults over time, increase social connection, and decrease their loneliness level. | Pre-post mixed-methods design | 15 | Older adults aged 65+  Mean age = 74 years; 6 (40%) female | PhotoSnake is a chat-based gaming app purposefully designed and developed by Games for Health. It aims to elicit interpersonal, meaningful, and playful interaction. Meaningful interaction refers to a feeling of social connectedness. The app consists of group chats where participants can send text messages and photos using their camera or photo archive. People in the same study group were in a chat group together. In PhotoSnake, someone sends a photo of an object, and the next person then sends a photo of an object, starting with the last letter of the object on the previous photo. The game was designed based on the assumption that the photos would trigger questions and story-sharing, thereby starting a conversation. | 3 sessions for 60-90 min 6 weeks | Loneliness: Six-item De Jong Giervald Loneliness Scale  Focus group interviews | Pre- and Post-test | At baseline, six participants were categorized as not lonely, three as moderately lonely, and six as severely lonely (Mdn = 2.0, IQR = 5.0). After the intervention (Mdn = 2.0, IQR = 4.0), compared with baseline, five people decreased, four increased, and five remained stable on the loneliness scale.  Loneliness did not change over time. They valued the in-person workshops to get to know each other. Lastly, inherent to a pilot study, we cannot conclude whether PhotoSnake improves social interactions. We did not have a control group or randomized conditions, so we cannot firmly conclude on the effectiveness on loneliness or social interaction. |
| Mixed technological interventions - Courses on the use of technical devices | In-Home Technology Training Among Socially Isolated Older Adults: Findings From the Tech Allies Program | Fields et al. | 2020 | Evaluating the program’s effect on participants’ loneliness, perceived social support, and technology use and identifying implementation and dissemination lessons for future interventions. | Pragmatic pilot trial | 57  IG: 27  CG: 30 | Older adults aged 60+  Mean age = 75 years; 30 (53%) female | IG: Digital traning sessions CTN created a learner booklet for each participant, outlining curriculum topics by week, including step-by-step visual guides and practice exercises. The eight sessions covered  (a) getting to know the iPad (hardware, touch screen, typing, voice dictation) (b) using the iPad (operating system, getting online, searching for information) (c) online safety (passwords, phishing, viruses) (d) email (creating an account, sending email, using the camera) (e) email safety (opening and replying to emails, identifying spam) (f) communicating via apps and FaceTime  (g) online communities (social media) (h) having fun and wrap-up (entertainment, learning, shopping)  CG: Wait-list (2 months) | 8 sessions  Weekly 8 weeks | Loneliness: Three-item UCLA Loneliness Scale  Social support: Subset of the Interpersonal Support Evaluation List (ISEL) | Pre- and Post-test | We found no change in loneliness from baseline (waitlist = 67% lonely, intervention = 92% lonely) to 2 months (waitlist = 66% lonely, intervention = 88% lonely) within either study arm (both arms: p = 1.0). Although we observed improvement in perceived social support over time within the intervention arm (baseline = 80% no perceived social support, 2-month = 62% no perceived social support), this change was not significant (p = .13), and we observed no change in the waitlist arm (baseline = 59% no perceived social support, 2-month = 50% no perceived social support, p = .63). Despite seeing no change in loneliness and perceived social support from survey data, the 15 post-training interviews uncovered a more nuanced picture of how participants’ feelings of connection related to improving technology skills and digital literacy. The intervention group showed no change in loneliness, marginally significant improvement in social support and technology confidence, and significant increase in technology use. Among the waitlist group, no changes were observed. Interviews showed some participants felt more connected to the world. |
| Mixed analogue interventions - Courses on the use of technical devices | Internet Programming to Reduce Loneliness and Social Isolation in Aging | Mullins et al. | 2020 | The purpose of this research was to describe program development and implementation and evaluation of the community-engaged, culturally informed IIS program to address social isolation and loneliness in senior living  community members. | Quasi-experimental | There were an average of 262 independent living residents | Older adults age 62 and older  Mean age males = 73.75 years   Mean age females = 75.25 years | Internet Information Station. Lessons were developed (e.g., computer basics, connecting with technology, open forum). Culturally informed internet health intervention to increase social support and decrease isolation and loneliness. The needs of the residents varied from low-skill level (e.g., how to turn a computer on) to higher-skill level (e.g., posting pictures on social media). | One year | Loneliness: R-UCLA Loneliness Scale | Two different intervals: the first was during the community assessment and before any health program interventions took place, and the second occurred after the technology course, between 4 and 6 weeks after the courses were complete. | Feelings of connectedness improved. The difference between groups on the total score for the scale was not statistically significant (p = 0.13), but the mean total score after the technology classes was lower than the mean at baseline. |
| Community interventions - Educational and Psychological interventions | Intervention effect of group reminiscence therapy in combination with physical exercise in improving spiritual well-being of the elderly | Ren et al. | 2021 | Exploring the intervention degree and improvement effect of group reminiscence therapy in combination with physical exercise on spiritual well-being of the elderly after the outbreak of the COVID-19 epidemic. | Randomized controlled study | 121  IG: 60  CG: 61 | Older adult aged 60+  59 (49%) female | IG: Psychological intervention based on group reminiscence. The themes of weekly group activities were set according to the feedbacks from group counseling, and the content of reminiscence therapy was taken as the main topic.  Taijiquan exercise, compiled by the General Administration of Sport of China, was selected as the program of physical exercise.  CG: Routine community health education  for 8 weeks. In the first, third, sixth and eighth week, the members of the control group participated in the “lecture on elderly mental health” given by members of the research team. The content covered daily life care, mental health care, prevention, and treatment of loneliness symptoms in the elderly as well as the methods to improve well-being of the elderly | 8 weeks | Loneliness: 8-item UCLA Loneliness Scale | Pre- and Post-test | There was no significant difference in the loneliness score between the two groups before the intervention (t = 0.082, P = 0.935); after one month of intervention, the loneliness score decreased in both groups, and the difference with their respective value before the intervention was statistically significant (P<0.001).  From the comparison of the loneliness score between the two groups after the intervention and the decrease in the loneliness score before and after the intervention, it can be seen that the effect was better in the experimental group than in the control group, and the difference was statistically significant (t = 2.008 and 0.047, P = 0.047 and 0.001, respectively). Before the intervention, there was no significant difference between the experimental group and the control group, but after 8 weeks of exercise intervention, the score of loneliness was lower in the experimental group than in the control group (P<0.05). |
| Multicomponent interventions - Befriending interventions | Intervention to reduce loneliness among older adults in the community: Making friends with volunteers | Hsu et al. | 2024 | This study aimed to examine the effects of providing a program that introduced volunteers to make friends with older adults to reduce loneliness and increase loneliness literacy. | One-group pretest–post-test experimental design | 87 | Aged ≥65 years  Mean age = 81.1 years  65–74 years = 15 (17.2%) 75–84 years = 44 (50.6%) ≥85 years = 28 (32.2%)  66 female (75.9%); 21 male (21 24.1%) | Leaders of the volunteers and the social workers were trained in loneliness literacy, skills of the questionnaire review,data collection, activity design and research ethics for data collection. Intervention activities included chatting and accompanying, arts and handicrafts, physical activities, and cognitive activities (table games, learning to use the internet, reading or classes.) Intervention program was designed by each community development association and delivered at the older adults’ homes. Two or three volunteers as a team were assigned to reach each older adult in the program and make friends with them. | 3 months | Loneliness: UCLA-6 Loneliness Scale | Pre- and Post-test | Loneliness was reduced (UCLA-6 Loneliness Scale scores decreased from 13.5 to 11.6), and loneliness literacy improved (loneliness literacy scores increased from 38.9 to 42.0). |
| Mixed analogue interventions - Trainer-administered | Intervention to reduce perceived loneliness in community dwelling older people | Rodríguez-Romero et al. | 2021 | Evaluating the reduction in perceived loneliness and depression and the increase in social support and quality of life in community-dwelling lonely people aged >65 years included in a community intervention compared with nonlonely controls from the same urban area. | Randomized controlled study | 55  IG: 29  CG: 26 | Older adults aged 65+  Mean age = 80.6 years; 48 (87%) female | IG: Intervention involving PHC nurses, family physicians, social workers and neighbourhood community agents. The intervention consisted of the following activities:  (i) health promotion and disease prevention with the aim of increasing the quality of life and well-being (ii) activities aimed at improving the mental and emotional state (iii) activities to create a social network among participants, teach some of the different neighbourhood resources and establish a common participative space for participants  CG: Care as usual | 18 sessions 6 months | Loneliness: UCLA Loneliness Scale   Social support: Duke-UNC-11 | Pre- and Post-test | There were significant improvements in the degree of loneliness in the intervention group, with 14 (48.3%) participants having no perceived loneliness at 6 months. In contrast, in the control group, a nonsignificant increase in patients with severe loneliness was observed at 6 months (7.7% preintervention vs. 19.2% postintervention). The mean improvement in the degree of loneliness was 8.63 points (95% CI 1.97–15.30) higher in the intervention group than in the control group (p = 0.012). The intervention increased social support. The percentage of participants with low social support was reduced from 37.9 to 13.8% (p = 0.002). In the control group, the results were very similar at baseline and at 6 months. Six months postintervention, 48.3% of the intervention group did not feel lonely compared with 26.9% of controls (p = 0.001). Social support (DUKE-UNC-11) increased from 33.5 +- 9.3 to 41.4 +- 6.6. |
| Religious intervention | Investigating the Effect of Religious Intervention on Mental Vitality and Sense of Loneliness Among the Elderly Referring to Community Healthcare Centers | Borji & Tarjoman | 2020 | Determining the effect of religious intervention on subjective vitality and sense of loneliness of the elderly referring to healthcare centers. | Semi-experimental study | 88  IG: 46  CG: 42 | Older adults aged 65+  IG: Mean age = 74.34 years; 25 (54%) female  CG: Mean age = 77.73 years; 22 (52%) female | IG: The religious interventions were performed according to the protocol of previously performed interventional studies. For this purpose, based on Quran verses and narrations of infallibles, the religious intervention program was implemented with major foci of recourse, gratitude, patience, forgiveness and amnesty, participation in public affairs, Dhikr, and pilgrimage.  CG: Usual care | 12 sessions of 30-45 min | Loneliness: UCLA Loneliness Scale (Version 3) | 4 measure points (before intervention, after intervention, 1 month after intervention, 2 months after intervention) | The findings show that according to repeated analysis of variance, the use of religious intervention has reduced the sense of loneliness of the elderly. Before intervention, there was no difference between mental vitality and fear of loneliness in the elderly, but after intervention, the amount of mental vitality increased and fear of loneliness decreased. |
| Mixed analogue interventions - Trainer-administered | “It makes me feel not so alone”: features of the Choose to Move physical activity intervention that reduce loneliness in older adults | Franke et al. | 2021 | (1) Evaluating whether an evidence-based PA intervention (Choose to Move; CTM) influenced PA and loneliness differently among self-identified ‘lonely’ versus ‘not lonely’ older adults  (2) Decribing factors within CTM components most likely to promote social connectedness/reduce loneliness. | Mixed-method study (quantitative, qualitative) | 452 | Older adults aged 60+  350 (77%) female | Choose to move (CTM) is a choice-based health promotion program that supports older adults with low levels of PA to become more physically active and socially connected. CTM consisted of: (i) a one-on-one consultation with an activity coach (ii) regular phone call ‘check-ins’ with an activity coach (iii) regular motivational group meetings with other CTM participants (up to 12 participants/ group), led by an activity coach. Group meetings included opportunities to share experiences, educational content on a specific topic and sharing of resources. | 6 months | Loneliness: 3-item UCLA Loneliness Scale   Social isolation: Three items adapted from two questions focused on frequency of contact | 3 measure points (baseline, mid-intervention after 3 months, post-intervention after 6 months) | Loneliness scores at baseline were significantly different between participants identifying as lonely and not lonely; this significant betweengroup difference was maintained at 3 and 6 months. Loneliness decreased significantly from 0 to 3 months in participants who identified as lonely at baseline; lower loneliness scores were maintained at 6 months (significantly different from baseline). There was no change in loneliness from 0 to 3 months in the ‘not lonely’ group. However, loneliness increased significantly in this group at 6 months compared to baseline. Loneliness decreased significantly from baseline to 3 and 6 months in participants identifying as lonely at baseline. Factors within CTM components that promote social connectedness/reduce loneliness include: Activity coach characteristics/personality traits and approaches; opportunity to share information and experiences and learn from others; engagement with others who share similar/familiar experiences; increased opportunity for meaningful interaction; and accountability. |
| Mixed technological interventions - Befriending interventions | Leveraging Public–Private Partnerships During COVID-19: Providing Virtual Field Opportunities for Student Learners and Addressing Social Isolation in Older Adults | Adepoju et al. | 2022 | Developing a virtual educational opportunity for students while also meeting the needs of vulnerable older adults in Houston, the third largest, and one of the most diverse cities in the U.S. | Mixed-Methods Study | 707 | Older adults aged 65+  Mean age = 72.33 years; 466 (66%) female | Pairing older adults with motivated college students to mitigate the detrimental effects of disconnectedness. Training included tips for active and reflective listening, as well as guidelines for introductions, incident escalation and referral to local resources and services. Referrals could include food pantries and other food distribution sites; meal-delivery services; financial assistance; housing support; or healthcare. Services were offered completely virtually, via telephone or audio-visual smart phone application, depending on the participants’ preference. | At least once a week 6 months | Loneliness: 3-item UCLA Loneliness Scale | Pre- and Post-test | At the beginning of the initiative, 87% of older adults reported loneliness, while 13% reported severe loneliness. Unadjusted post-measurement reports revealed some reductions in loneliness; the proportion of older adults who reported loneliness decreased from 84.2% to 40%, the proportion of severely lonely older adults reduced from 15.8% to 12.4%, while 48% of the sample reported no longer feeling lonely. Findings include student/learner outcomes, as well as self-reported improvements in loneliness scores, and unhealthy physical and mental health days among enrolled older adults. |
| Mixed analogue interventions - Courses on the use of technical devices | Loneliness and Social Engagement in Older Adults Based in Lombardy during the COVID-19 Lockdown: The Long-Term Effects of a Course on Social Networking Sites Use | Rolandi et al. | 2020 | Investigating whether older adults trained for SNSs use showed more frequent use of SNSs and increased resilience (reduced loneliness and social isolation) during the period of forced self-isolation due to the COVID-19 outbreak. | Quasi-experimental study | 130  IG: 60  CG: 70 | Older adults aged 80+  Mean age = 81.8 years; 68 (52%) female | IG: SNS courses The themes covered were: smartphone use, Facebook and WhatsApp use, privacy rules, and fraud risk prevention using Facebook. Participants were provided with user-friendly smartphones designed for older adults.  CG: Wait-list | 5 sessions Twice a week 1 year | Loneliness: 3-item UCLA Loneliness Scale  Social engagement: 6-item Lubben Social Network Scale (LSNS-6) | Pre- and Post-test | When longitudinal analyses were performed to test the impact of SNSs training on one-year change in the measures of interest, a slight reduction in social engagement was found in the trained group, on both LSNS-6 Total (F(1) = 7.85, p = 0.006) and LSNS-6 Family (F(1) = 8.16, p = 0.005). No significant differences between groups were found for the UCLA scale and single items using a generalized linear model. Participants trained for SNSs use reported significantly higher usage of SNSs and reduced feeling of being left out. Compared to pre-lockdown levels, individuals trained for SNSs use showed a lighter reduction in social contacts. |
| Mixed Technological intervention - Synchronous group intervention and exercises | Loneliness Decreased for Older Adults Using Telephone and Video Communications | Mierzwicki et al. | 2023 | Studying feasibility and impact of telephone and video communication on perceptions of loneliness and social isolation in community-dwelling older adults | Quasi-experimental interventional research study | 16 | Older adults agd 65+  Mean age = 78.86 years; 11 (69%) female | Engagement of each participant in a weekly Zoom or telephone phone call anchored by prepared conversation topics and exercices. Conversation topics: (1) Ice breakers, introductions, background (2) Travel (3) Life Accomplishments (4) Family and friends (5) Childhood memories (6) Current favorites/thoughts on technology (7) Advice to younger self/advice for future health professionals  (8) Program reflection/reassessment Exercices: (1) Lower body (2) Upper body | Conversations of 45-60 min One time per week 8 weeks | Loneliness: UCLA-3 Loneliness Scale, UCLA-20 Loneliness Scale | Pre- and Post-test | The UCLA-20 demonstrated statistical significance (p = .017) for the full cohort with large effect size (Hedges' g = 1.273). UCLA-3, SFS-8 and FRAIL scale did not demonstrate statistically significant pre-post differences. No statistically significant differences (p > .05) were identified between the telephone and video communication groups. |
| Individual interventions - Trainer-administered | Meditation program mitigates loneliness and promotes wellbeing, life satisfaction and contentment among retired older adults: a two-year follow-up study in four South Asian cities | Pandya | 2021 | Examining the impact of a meditation program in mitigating loneliness and promoting wellbeing, life satisfaction and contentment among retired South Asian older adults. | Randomized controlled study | 323  IG: 166  CG: 157 | Older adults aged 60+  62-68 years | IG: Classes meditation program The meditation repertoire of relaxation, slowness and innter watchful awareness was designed as a social cognition influencing and loenliness-mitigating intervention. The lesson comprised of the following repertoire: (1) centering in silence (2) instant relaxation through isometric contraction of muscles in supine position (3) tree posture (4) half-wheel posture (5) forward-bending posture (6) backward-bending posture (7) deep relaxation and slow breathing in a comfortable sitting position involving silence, concentration and centering (8) deep relaxation and slow breathing in a supine position involving silence, concentration and centering (9) closing moments of silence  CG: No intervention | Sessions for 45 min Once a week 2 years | Loneliness: De Jong Giervald Loneliness Scale | Pre- and Post-test | There was no significant difference in the post-test outcome measure scores of the control group (p = .08-.90; d = 0.01-0.18). Post-test DJGLS-6 scores of the intervention group were higher than the control group (mean difference = 2.48, p = .001, d = 2.43) and their own pre-test scores (mean difference = 2.54, p = .001, d = 2.47). There were significant mean differences in the post-test scores on loneliness, wellbeing, life satisfaction and contentment outcomes of the intervention group, with high observed effect sizes (Cohen's d range = 2.43-8.78, p < .01). The intervention grroup older adults reported that they were less lonely and experienced greater wellbeing, life satisfaction and contentment post-test (n^2 = .71-.78, p < .01) |
| Web-based interventions - Individual interventions | Mental Health and Psychosocial Support Online-Based Services To Improve Elderly Integrity and Reduce Loneliness During the Pandemic Covid-19 | Marliana et al. | 2022 | Identifying the incident of mental-emotional disorder, level of loneliness, the achievement of psychosocial developmental tasks through mental health and psychosocial support (MHPSS). | Quasi-experimental pre-post-test with a control group | 106  IG: 54  CG: 52 | Not mentioned | IG: Online MHPSS  MHPSS is a form of support provided to protect or improve psychological well-being or prevent or treat mental health conditions.  CG: Care as usual | Daily 8 weeks | Loneliness: De Jong Giervald 11-item instrument | Pre- and Post-test | The elderly with severe loneliness decreased from 31.5% to 1.8%, and the elderly with moderate loneliness (22.3%) felt less lonely (75.9%). There is a significant influence between the level of loneliness on the intervention group and control group, and it can be seen from the value of p =0.000 (p less than 0.05). Regression analysis showed a significant difference in the decreasing mental-emotional disorder and level of loneliness in the intervention group (p value≤ 0,005) compared to the control group. |
| Multicomponent interventions - Web-based intervention with subjective physical activities | Mitigating Feelings of Loneliness and Depression by Means of Web-Based or Print-Based Physical Activity Interventions:  Pooled Analysis of 2 Community-Based Intervention Trials | Lippke et al. | 2022 | This study aimed to compare the effects of web- and print-based PA interventions on self-reported PA, stage of change, determinants of PA, loneliness, and depression. | Randomized intervention study | 831  Web-based PA intervention, or WLCG = 589   Print-based PA intervention = 242 | Adults aged ≥60 years  Gender not mentioned | IG 1: Print-based intervention group; web-based intervention with subjective PA self-monitoring, web-based intervention with subjective and objective PA self-monitoring or WLCG --> engage in physical exercises  IG 2: Web-based intervention group; print-based intervention with subjective PA self-monitoring via a printed PA pyramid and web-based intervention with subjective PA self-monitoring via a web-based PA pyramid in the form of a booklet  CG: Wait-list control group  PA intervention aimed to improve overall self-monitoring capabilities regarding PA and enable transfer for the time after the intervention. | 10 Weeks | Loneliness: Used the item "I felt lonely" from the CES-D scale (Center for Epidemiologic Studies Depression) | T0 and T1 | WLCG increasing in its loneliness, the web-based IG maintaining its previous level, and the print-based IG decreasing in its loneliness over time. The web-based and print-based interventions improved loneliness, and depression compared with the control condition. None of the interventions had a significant direct effect on loneliness compared with the WLCG (web-based: c′ path −0.01, 95% CI −0.11 to 0.08, SE 0.05; print-based: c′ path −0.09, 95% CI −0.24 to 0.06, SE 0.08). |
| Videoconferencing interventions - Synchronous exercises (Group or individual) | Moving together - benefits of an online dance program on physical and mental health for older women: an exploratory mixedmethod study | Hansen et al. | 2024 | This exploratory study investigated the benefits of a 12-week online dance training intervention on mental and physical health outcomes for older women. | Exploratory mixed-method study | 32 | 65 years or older  Mean age = 74.0 years; all female | Two weekly 60-min classes of improvisation and salsa delivered online through video call applications. Led by professional dance instructors experienced with online teaching and trained in inclusive dance practice. Delivered online through video calls using the Zoom meeting application. Participants could choose to attend the online classes from home (alone) or at one of four activity centers in the municipality (groupbased). | 12 weeks of two weekly classes of 60 min training | Focus group interviews   Loneliness and social isolation: UCLA Loneliness Scale | Prior to and after 12 weeks | No significant changes were reported in HRQOL and loneliness from the quantitative data, although the qualitative data did reveal improved feelings of physical health and wellbeing. The qualitative results suggest that when implemented in a community setting with mutual participation by others, online dancing may positively influence social engagement. |
| Community interventions - Group meetings | “Not Alone in Loneliness”: A Qualitative Evaluation of a Program Promoting Social Capital among Lonely Older People in Primary Health Care | Coll-Planas et al. | 2021 | We aimed at exploring participants’ experiences of loneliness and participation prior to the program and its perceived benefits. | Qualitative study | 38 participants   26 older people who participated in the program, nine older volunteers and six health and social care professionals | Participants = 65 - 80 years; 37 female | Promoting peer support and participation in community assets. Peer support was promoted through sharing opinions and experiences around loneliness and participation prompted by a diversity of pictures. They visited and experienced activities in five local community assets to promote their engagement in these settings. | Sessions were one and half hours long and took place once a week during 15 weeks. | Interviews and focus groups | One time-point | According to participants, the group provided companionship, a feeling of social integration and sense of belonging to the group. Most of the participants reported that their loneliness decreased after the program by feeling accompanied by peers and professionals. While some people said they no longer felt lonely because of new friendships, others continued to suffer from loneliness, but with less intensity. |
| Robot interventions - Pets | Older Adults’ Experiences in a Web-Based Intervention for Loneliness | Hudson et al. | 2023 | Explore the experiences of vulnerable older adults in a web-based loneliness intervention. | Qualitative study | 24 | Mean age = 73 years; 11 females | Web-based loneliness intervention.  Cognitive-behavioral, acceptance, mindfulness, and positive psychology concepts were adjusted into easy-to-understand digital materials. | Average = 78 days (11weeks) | Semi-structured interviews | One time-point | Participants gaining new social skills, improved relationships, and increased confidence to initiate and maintain social contact. Participants reported numerous positive outcomes related  to increasing their social activity and alleviating loneliness. |
| Web-based interventions - Group intervention | Web-based interventions - Group interventions | Gusdal et al. | 2023 | The aim of this study was to explore older adults’ use of a new, co-designed and research-based web platform for social interaction from the perspectives of older adults, researchers, and social services personnel. | Mixed Methods Study | 20 | Age 60+  Mean age = 77 years (63–93); 18 female | Web platform for social interaction “the Fik@ room”. The Fik@ room consisted of digital coffee/Fik@ tables. The conversations included either video, voice, or chat conversations about topics of the older adults’ own choice. The Fik@ room also included a bulletin board where older adults could post messages and schedule meetings in the Fik@ room or at a physical location. | Eight weeks | Loneliness: UCLA Loneliness Scale (Version 3)  Social activities and contacts and their satisfaction with them: Social network questionnaire | Pretest, during the test, and posttest | It enabled to expand social network and develop new friendships, but experiences of loneliness were not reduced.The results showed no significant difference between the pretest and posttest. |
| Web-based interventions - Group interventions | Online acceptance and commitment therapy as treatment for loneliness among older adults: Report of a pilot study | Zarling et al. | 2023 | The aim of this study was to pilot test a novel ACT-based online intervention to decrease loneliness in older adults living in the community. | Quasi-experimental design | 193 | Aged 65 or over  Mean age = 70.20 years (66 to 95); 61.6% female | IG: Acceptance and commitment therapy program consisting of eight interactive modules on a  digital platform, designed to teach participants skills to address common contributors to loneliness  CG: No intervention | 8 weeks | Loneliness: Short 10-item version of the UCLA Loneliness Scale | Pre-intervention, postintervention, and at a one-month follow-up | Average levels of loneliness significantly decreased from pre- to posttreatment, b =- 0.013, t (385) = -4.69, p < 0.001 (Cohen's d = 0.30). The improvement in loneliness was maintained at the one-month follow-up assessment. The change was significantly greater than that observed in members of a “hold-out” group of lonely individuals that did not participate in the intervention (Cohen's d = 0.24). |
| Individual interventions - Trainer-administered | Pawsitive Connections: The role of Pet Support Programs and pets on the elderly | Cryer et al. | 2021 | This study aimed to describe the experiences of people over 65 who utilise PSPs in Melbourne, Australia. | Qualitative description study | 18 14 Participants 4 PSP providers | Mean age = 81 years (65–90); 12 female | Pet Support Programs (PSPs) e.g. the PSP assists the elderly to look after their pet. | Not mentioned | Interviews with 14 PSP clients, and key informant interviews with four PSP providers. | One time-point | Social isolation was reduced by the PSP through access to social support groups, as well as the provision of a regular visitor who provides meaningful conversation and companionship. |
| Community interventions - Group meetings | Playing Kioku Reduces Loneliness in Older Adults: A Pilot Study | Bodner et al. | 2024 | Examine the effect of the Kioku board game on well-being outcomes. | Pilot Study | 151  IG: 72 CG: 79 | Older adults Mean age = 75.05 years  IG: Mean age = 76.83 years; 87.5 % feamle  CG: Mean age = 73.42 years; 89.9 % female | IG: Kioku enable small group interactions with a focus on encouraging participants to create stories through mutual attention and interaction. Groups of 4-5 players , chose a cube word and narrated a story. Kioku is a storytelling board game that enhences social conncetions and cognitive engagement.   CG: Wait-list (engaged in continous regular activities at the same-day centers in which the intervention took place. | Weekly intervention for 12 weeks | Loneliness: UCLA Lonliness Scale (revised shortendes 8-item version) | Baseline and at 12 weeks | Loneliness descreased in theintervention group (mean difference: 0.62 pionts, M = 2.24, SD = 0.48 in T1; M = 1.62, SD = 0.39 in T2; t(71) = 13.59, P < 0,001, Cohens d = 1.60), and increased in the control group (mean difference: 0.18 points, M = 1.92, SD = 0.58 in T1; M = 2.10, SD = 0.56 in T2; t(78) = - 5.36, P < 0.001, Cohens d = -0.60). Kioku board game intervention decrease loneliness and promoting well-being. |
| Individual interventions - Self-administered | Promoting Connection in the Faith Community Through Letter Writing | Long et al. | 2023 | The intervention was initiated to evaluate the degree of loneliness within the faith communities of nursing students and to evaluate if participation in the letter writing initiative decreased levels of loneliness and promoted connection among older faith community members. | Quasi-experiment design | 34 | 78 to 86 years old; all female | Undergraduate nursing students who were matched with members of their own faith community wrote letters to older adults. | 10 weeks | Loneliness: UCLA Loneliness Scale (Version 3) | Pre- and Post test | Results demonstrated a significant decrease (t = 0.0126099, p = .005) in the level of loneliness pre-intervention (M = 20.6, SD = 12) and post-intervention (M = 15.9, SD = 11.7). |
| AI-based interventions | Reducing Loneliness Among Aging Adults: The Roles of Personal Voice Assistants and Anthropomorphic Interactions | Jones et al. | 2021 | This study aimed to understand the influence of a PVA on loneliness reduction among adults of advanced ages, i.e., 75+, and explore anthropomorphism as a potential underlying mechanism. | Single-group quasi-experimental study design | 16 | Adults 75 years of age and older   Mean age = 85.2 years (77 to 96); 69% female | Amazon Echo PVA (personal voice assistants) | 89 weeks | Loneliness: eight-item UCLA loneliness Scale | Before the study (baseline), after 4 weeks (week 4) of use | Reported significantly lower loneliness (baseline mean = 2.22, SD = 0.42; week 4 mean = 1.99, SD = 0.45, Z = −2.45, and p = 0.01). Anthropomorphic interactions with the Alexa PVA will predict reductions in loneliness among aging adults living alone in the first 4 weeks of the Alexa PVA intervention. |
| Robot interventions - Pets | Reducing loneliness and improving well-being among older adults with animatronic pets | Tkatch et al. | 2020 | Determine the feasibility of an animatronic pet program, and whether ownership of animatronic pets would decrease loneliness and improve well-being among lonely older adults. | Experimental study | Completed T1: 216 Completed T1 and T2: 168 Completed T1, T2, and T3: 125 | Mean age 65–74 years, T1: 51.4%  T2: 48.2% T3: 49.6%, 52.8% female   Mean age 75–84 years, T1: 30.6%  T2: 31.5% T3: 29.6%, 55.4% female  Mean age > -85 years, T1: 18.1% T2: 20.2% T3: 20.8%, 56.8% female | Animatronic pet of their choice (cat or dog) | 60-day period | Loneliness: 10-item short version of the UCLA Loneliness Scale | T1 survey at the receipt of the pet,  T2 survey delivered approximately 30 days after they had received the pet and a T3 survey approximately 60 days after receipt of the pet | Post survey data indicated that loneliness decreased, while mental well-being, resilience, and purpose in life improved. Greater time spent interacting with the pets was associated with an improvement in participants’ mental health. |
| Videoconferencing interventions - Synchronous individual interventions | Remotely Administered Resilience- and Wisdom-Focused Intervention to Reduce Perceived Stress and Loneliness: Pilot Controlled Clinical Trial in Older Adults | Jeste et al. | 2022 | Aiming to reduce perceived stress and loneliness in community-dwelling older adults. | Multiple-phase-change single-case experimental design | 20 | Adults >65 years  Mean age = 78.3 years, 80% female | IG: Six one-hour individual-level sessions conducted remotely once-a-week by research staff. Multi-pronged intervention:  1) Cognitive: education about empathy, compassion, self-compassion, loneliness, and attitudes toward aging; and CBT (Cognitive Behavioral Therapy)-informed. 2) Affective: role plays; mindfulness exercises and meditations; and self-compassion exercises. 3) Behavioral: savoring and gratitude practices including a daily gratitude diary; social skills training.  HIPPA-compliant teleconferencing platform.  CG: Each participant serve as their own control | Three successive 6-week phases: control, intervention, and follow-up periods. The intervention included six once-a-week one hour sessions. A) control period (weeks 0-6), B) intervention period (weeks 6−12), and C) follow-up period (weeks 12-18) | Loneliness: 20-item UCLA-3 Loneliness Scale | Weeks 0 (beginning of control period), and end of weeks 6 (end of control), 12 (end of intervention), and 18 (end of follow-up) | Reduction in perceived stress and loneliness, and increase in resilience, happiness, and components of wisdom and positive perceptions of aging. During the follow-up period (week 12 vs. week 18) there were no meaningful changes. |
| Videoconferencing interventions - Synchronous individual interventions | Remotely-administered resilience and self-compassion intervention targeting loneliness and stress in older adults: a single-case experimental design. | Patapoff et al. | 2023 | Aimed to reduce loneliness and stress by increasing prosocial behaviors and enhancing levels of resilience and self-compassion. | Multiple-phase-change single-case experimental design | 43 | Age ≥65years  Mean age = 76.9 years, 77% female | IG: 6-week remotely-administered resilience and self-compassion intervention using techniques from cognitive behavioral therapy and resilience training.  Intervention sessions were conducted via Zoom, a video-conferencing platform.Technology-Assisted Compassion Training for Seniors (T-ACTS), a manualized positive psychiatry intervention  CG: Participants served as their own control | Three consecutive 6-week phases. Phase one was the control period (baseline-week 6), phase two was the intervention period (weeks 6-12), and phase three was the follow-up period (weeks 12-18).Weekly 60-minute one-on-one virtual sessions with a trained facilitator. | Loneliness: UCLA Loneliness Scale | Prior to the control period (baseline), before intervention (week 6), after intervention (week 12), and after follow-up period (week 18) | Participants reported a reduction in loneliness (p=0.003; ηp 2=0.18) from control to intervention completion (weeks 0−12). Loneliness reduced significantly during the control phase (weeks 0−6). Findings indicate that remote resilience and self-compassion intervention for older  adults targeting loneliness and chronic stress was efficacious. |
| Robot interventions - Pets | Robotic Pet Use Among Community-Dwelling Older Adults | Hudson et al. | 2020 | The primary purpose of this study was to explore the efficacy of robotic pets in alleviating loneliness for older adults. | Qualitative study | 20 | 65 years of age  Mean age = 67 years; 10 females | Robotic pet, (interactive voice support) phone call encouraging them to interact with the pet. Robotic pet offered several interactive features. | 60 days | Semi-structured interviews | Three post surveys were administered (upon receipt of the pet as well as 30 and 60 days later) | Participants living alone, with fewer social connections and less active lifestyles, derived the most benefit from interacting with their pets. Robotic pets may be an effective solution for alleviating loneliness in older adults, especially among those who live alone, have fewer social connections, and live less active lifestyles. |
| Individual interventions - Self-administered | Self-affirmation training can relieve negative emotions by improving self-integrity among older adults | Dang et al. | 2023 | Intended to use selfaffirmation intervention to relieve negative emotions of older adults and to explore the role of self-integrity in this process. | Exprimental study | IG: 88  CG: 85 | Age ≥ 60 years  IG: Mean age = 65.26 years; 53 female  CG: Mean age = 64.14 years, 46 female | IG: Self-affirmation intervention group (Each participant received one experimental condition and individually completed experimental manipulation in the community activity room)  CG: Three different experimental manipulations (differs from the intervention group) | Three intervention session for three weeks | Loneliness: Short version of the University of California, Los Angeles (UCLA) Loneliness Scale | Pre- and Post Test | The main effects of group for loneliness were significant, F (1, 170) =18.82, p < .001, ηp 2 = .100, respectively. Loneliness of the intervention group was lower than those of the control group. Self-affirmation intervention boosted self-integrity and reduced negative emotions, including loneliness. |
| Community interventions - Art and music interventions | Sing4Health: Randomised controlled trial of the effects of a singing group program on the subjective and social well-being of older adults | Galinha et al. | 2021 | Aims to fill this gap by analysing the effects of a 34-session singing group programme (SGP) on participants' subjective and social well-being and the mediating roles of social identification with the singing group and of self-esteem. | Randomised controlled study, mixed method study | IG: 89  CG: 60 | Mean age = 76.66 yeears  IG: Mean age = 76.76; 77 female   CG: Mean age = 76.51 ; 47 female | IG: Sing4Health programme (relaxation and vocal warm-up exercises, vocal technique, rehearsal of repertoire, a social component, creation and presentation of a final show)  CG: Active waiting-list control (usual social, artistic and leisure activities) | IG: 34 two-hour sessions, two times per week  CG: All activities shorter than 3 h per week | Social Identification: Four Item Measure of Social Identification FISI   Loneliness: UCLA Loneliness Scale  Social Identification: Four-Item Measure of Social Identification | 2 weeks before and after the SGP (T0 and T1) and in a 6-month follow-up (T2) | Significant effects of the SGP on the positive affect, social well-being and marginally on the self-esteem of the participants. Well-being benefits, including responses such as feeling good, feeling better, forgetting problems, feeling pride, feeling enthusiasm, feeling affection or having a good experience. A significant reduction in loneliness in the IG, between T0 and T1, was observed and sustained in the follow-up it was not significantly different from the WLG. |
| Application-based intervention | Social and physical effects of a pedometer and communication application among older men: a mixed-methods, pre/post pilot study | Abe et al. | 2023 | Aimed to clarify the effects of Kikoeru on social connectedness related to interactions through the app, subjective health, loneliness, and setting a target number of steps. | Pilot mixed method study | 7 | Mean age = 74 ± 4.0 years; all male | ‘Kikoeru’, a pedometer and communication application (app) with functions that include voice conversations and messages, photograph sharing, and pedometer sharing among user. | Two months | Loneliness: Ando-OsadaKodama loneliness scale | Self-reported questionnaire before and after the intervention. | Loneliness decreased for four out of seven people, remained the same for three, and did not worsen for anyone.  Participants experienced social connectedness, reduced loneliness. Although no significant difference was found, after the intervention. Older men could maintain interpersonal relationships and create social connectedness both online and offline. |
| Mixed analogue interventions - Courses on the use of technical devices | Social media and social wellbeing in later life | Quinn | 2021 | Examine the potential social effects of social media use among older adults. | Randomized controlled study | IG: 17  CG: 19 | IG:  Mean age = 78.00 years; 13 females   CG: Mean age = 77.00 years; 12 females | IG: Four-week social media training workshop; instructional workshops were devoted to helping participants learn how to use the social media platforms of Facebook and Twitter  CG: Wait list | Once per week for four weeks, two hours per session | Loneliness: UCLA Loneliness Scale, Version 3  Social connection: the social connectedness scale   Perceptions of social integration: Social integration sub-scale of the social provisions scale | At baseline ( just prior to the start of the workshop), at four weeks (completion of the social media intervention workshop for the intervention group) and at four months (three months after workshop completion for the intervention group) | There was no significant interaction between the intervention and time (F2,68 = 0.75, p = 0.48, ηp 2 = 0.02) and no significant main effects for the intervention (F1,34 = 1.00, p = 0.32, ηp 2 = 0.03) in reported feelings of loneliness. Both groups experienced significant improvements (decreases) in perceived loneliness over the study period (F2,68 = 61.58, p <0.001, ηp2 = 0.64). |
| Mixed analogue interventions - Trainer-administered | Status Quo or drop-off: Do older adults maintain benefits from choose to move-a scaled-up physical activity program-12 months after withdrawing the intervention? | McKay et al. | 2021 | Evaluate whether Choose to Move participants maintained their intervention-related gains in physical activity (PA), mobility, and social connectedness 12 months after the intervention ended. | Effectiveness implementation study design | 223 | 181 female (77%)  Age group 60–74 years: 166 (71%) Age group ≥75 years: 69 (29%) | Choose to move. : (1) a one-on-one consultation, and (2) regular phone call ‘check-ins’-with an activity coach, (3) regular motivational group meetings (groups) with other CTM participants (up to 12 participants/group), led by an activity coach. CTM activity coaches provided personalized support to create PA action plans customized to each individual’s activity preferences, resources, and mobility capacity. Provided opportunities for social support and for older adults to share and learn with fellow participants. | 6-month intervention | Loneliness: UCLA 3-item questionnaire | 0 months (baseline), 6 months (end of intervention), and 18 months (12-months postintervention) | Intervention-related benefits in loneliness, social isolation, mobility, and muscle strength were maintained between 6 and 18 months in the younger participants. Older participants maintained their intervention benefits in loneliness, mobility, and muscle strength. When compared with baseline values,  social isolation increased. The loneliness scores at 18 months were not significantly different from the baseline values in either the younger or older participants. |
| Videoconferencing interventions - Synchronous group interventions | Teaching and practicing cognitive-behavioral and mindfulness skills in a web-based platform among older adults through the COVID-19 pandemic: A pilot randomized controlled trial | Shapira  et al. | 2021b | Explored the effects of a short-term digital group intervention aimed at providing cognitive behavioral and mindfulness tools and skills to reduce loneliness and depression and to increase social support among older adults. | Pilot randomized controlled study | IG: = 64   CG: 18 | IG: Mean age = 72.1 years, 81% female (52)  CG: Mean age = 71.7 years, 78% female (14) | IG: Zoom videoconferencing platform, groups of 5–7 people between 1–1.5 h;  (a) guided group discussions, and (b) learning and practicing CBT techniques and skills (e.g., breathing, guided imagery of a ‘safe place’, constructing positive self-talk, mindfulness meditations, and more)   CG: Wait-list (no treatment) | Seven online sessions, over 3.5 weeks | Loneliness: UCLA Short Scale for Measuring Loneliness  Social support: Duke-UNC Functional Social Support Questionaire | Baseline, immediately post-intervention, and at 1-month follow-up | Loneliness levels significantly decreased post-intervention, this benefit was not maintained at one-month follow-up. Significant reductions in depression in the intervention. Social support did not significantly change following the intervention. |
| Application-based interventions | The Benefits of Technology for Engaging Aging Adults: Findings From the PRISM 2.0 Trial. | Czaja et al. | 2024 | This study evaluated the Personal Reminder Information and Social Management 2.0 (PRISM 2.0) software, in terms of enhancing social engagement and quality of life, and decreasing loneliness among older adults. | Randomized field trial | 245 | Aged 64 to 99 years  78% female  Senior housing: Mean age = 74.42 years   Rural locations: Mean age = 73.70 years   ALC: Mean age = 85.77 years | IG: PRISM 2.0 (integrated software system designed for aging through an iterative design process). PRISM 2.0 was an Android application. Email feature designed to support connectivity and communication; Internet access; a Classroom feature intended to augment procedural knowledge and provide new learning opportunities; a  Socializing feature that provided ways to increase socialization and engage in activities such as volunteering and mentoring; a Health feature that included information on health conditions, health-related apps; vetted health resources, and a health glossary in simple language; a Community feature that included vetted lists of national and local resources and community events; a Games feature that included single and multiplayer interactive web-based games; a Calendar feature that included a reminder functionality; a Videochat feature, Skype; and online help. online help   CG: Standard Tablet (without PRISM). Same amount of contact and training as those in the PRISM 2.0 condition. | 6 months | Loneliness: UCLA Loneliness Scale  Perceived social support: MOS Social Support Survey  Social connectedness: Social Connectedness Scale | Baseline and 6 and 9 months postrandomization | Participants in rural locations and senior housing in both conditions reported less loneliness and social isolation, and greater social support and quality of life at 6 months, and an increase in mobile device profciency (b = −0.69, 95% CI [−1.20, −0.18], t(76) = −2.66, p = .008) (b = −1.02, 95% CI [−1.48, −0.56], t(75) = −4.38, p < .001). Technology applications may help reduce social isolation, loneliness, and enhance quality of life. There was no change in loneliness (b = −0.23, 95% CI [−0.79, 0.33], t(64) = −0.79, p = .43) for those in ALCs. Participants in senior housing and in rural locations reported a signifcant improvement in quality of life and social support over time. For those in ALCs, there was no change in quality of life over time and social support. Participants in senior housing across both conditions reported signifcant improvements in health-related quality of life over time. The other groups did not report any changes. There were signifcant reductions in social isolation among participants in senior housing and in rural locations in both conditions over time but not for those in ALCs. |
| Community interventions - Group meetings | The Effect of a Reminiscence Therapy-Based Hybrid Board Game on Anxiety and Loneliness Levels in Older Adults: An Experimental Study | Liu et al. | 2024 | Objective is to empirically validate the effectiveness of a reminiscence therapy-based hybrid board game, Journey of Memories, in reducing anxiety and loneliness among older adults. | Randomized controlled study | 38  IG: 20  CG: 18 | Between 61 and 75 years  Mean age = 67.16 years; 60.5 % female (23) | IG: Three sessions of the Journey of Memories hybrid board game intervention, with a 5-day interval between each session.   CG: No intervention | 12 days | Loneliness: UCLA Loneliness Scale and in-deph interviews | Beginning (pre-test) and the end (post-test) | 20 participants in the experimental group showed significant reductions in loneliness levels (UCLA Loneliness Scale [UCLA] average scores decreased from 39.11 to 36.11, P = 0.002). Loneliness score of the experimental group was siginficant lower than that of the control group (P=0.034). |
| Community interventions - Art and music interventions | The Effect of Group Art Therapy on Loneliness and Hopelessness Levels of Older Adults Living Alone: A Randomized Controlled Study | Aydin & Kutlu | 2021 | Aimed to test group art therapy using clay as a medium as a method to cope with feelings of loneliness and hopelessness in older adults. | Randomized controlled study | IG: 30  CG: 30 | IG: Mean age = 72.60 ± 8.10 years; 76.7% female (23)  CG: Mean age = 72.53 ± 7.75 years; 80.0% female (24) | IG: Six 1.5-hour weekly sessions of group art therapy using clay, and interviewed face-to-face twice  CG: Interviewed face-to-face twice | Weekly clay based group art therapy sessions of 60–90 minutes were carried out for six weeks. | Loneliness: UCLA Loneliness Scale (UCLA-LS)   Hopelessness levels: Beck Hopelessness Scale (BHS) | Beginning (pre-test) and the end (post-test) | Significant difference was found between the pre-test and post-test scores of the IG and CG. Clay-based group art therapy can contribute toward decreasing the sense of loneliness and hopelessness. |
| Community interventions - Group meetings | The Effectiveness of a Thanks, Sorry, Love, and Farewell Board Game in Older People in Taiwan: A Quasi-Experimental Study | Chen & Tsai | 2022 | Examine the effectiveness of the four themes of life board game to enhance interpersonal communication, interpersonal relationships, and self-efficacy and decrease loneliness. | Quasi-experimental study | IG: 46  CG: 45 | IG:  70 years and below = 4; above 70 years = 42; 40 female, 6 male   CG: 70 years and below = 2; above 70 years = 43; 38 female, 7 male | IG: Played the four themes of life board game for 4 weeks  CG: Participated in routine community center activities | 90 min each for a total of 4 weeks | Interpersonal Communication: Interpersonal Communication Scale  Interpersonal Relationship: Interpersonal Relationship Scale  Loneliness: UCLA Loneliness Scal (v3) | 3 measure points (Baseline (T0), at the end of the intervention (T1), and at 3 months after the end (T2)) | IG had statistically significantly larger improvements in scores on interpersonal communication, self-efficacy, and loneliness at 3 months after the end of the intervention. |
| Videoconferencing interventions - Synchronous group intervention | The effectiveness of facilitator-led remote interactive intervention for loneliness, quality of life, and social support among seniors in communities during the COVID-19 pandemic: a randomized controlled experiment | Liu et al. | 2023 | Investigated the impact of a 12-week remote interaction intervention on loneliness, quality of life, and social support for seniors living in a community during the COVID-19 pandemic. | Randomized controlled study | IG: 50  CG: 50 | IG: mean age = 72.90 years; 37 (74%) female participants   CG: mean age = 72.78 years; 35 (70%) female participants | IG: Bidirectional remote interaction intervention. The online interactive interventions were conducted with a facilitator conducting live classes and alternating between static and dynamic classes. Static classes included instruction on how to use smartphones and apps, creating art, appreciating music, and disseminating knowledge related to epidemic prevention. The dynamic courses included aerobics, stretching, balance training, mouth exercises, and acupressure.  CG: Unidirectional remote interaction intervention | 12-weeks | Loneliness: UCLA Loneliness Scale (UCLA)  Social support: Inventory of Socially Supportive Behavior (ISSB) | Pre-test one week before the start of the intervention and a post-test one week after it ended | Intervention group scored significantly higher than the control group on the WHOQOL-BREF in the physical health and social relationships domains after the intervention.  There was no significant difference in loneliness scores between the intervention and control groups. Seniors can improve their quality of life using social apps. |
| Community interventions - Educational and Psychological interventions | The Effectiveness of Social Participation Educational Program on the Feeling of Loneliness of Elderly People in Rural Areas of Baiza City (South of Fars Province) | Nazari et al. | 2021 | Determine the impact of the educational program of social participation on the feeling of loneliness. | Quasi-experimental study | 239  IG: 119  CG: 120 | Elderly people aged ≥60 years  IG: Mean age = men 46±38.70 years, women 73±61.3 years; 63 female  CG: Mean age = men 57±47.50 years, women 63±52.5 years; 73 female | IG: Social participation training program (e.g. empowering people using  emotion-expressing techniques and developing optimism and positive thinking; prioritizing social participation through question-and-answer methods)  CG: No invervention | Five weeks through five 60-80 minute educational sessions | Loneliness: UCLA Loneliness Scale | Before-and-after intervention | Mean score of social participation increased in the intervention group from 6.17±3.86 to 17.98±3.84 after the intervention. Improvement in loneliness feeling  scores of the mentioned group form 62.24±.7.53 to 28.86±6.88 after the intervention (P<0.001). The findings of this study showed a significant relationship between social participation and the feeling of loneliness. |
| Videoconfonferencing interventions - Synchronous exercises | The effectiveness of synchronous tele-exercise to maintain the physical fitness, quality of life, and mood of older people - a randomized and controlled study. | Zengin Alpozgen et al. | 2022 | Aims to investigate the efects of a synchronous tele-exercise program on physical ftness, quality of life, loneliness, and mood change under social isolation during the coronavirus pandemic. | Randomized controlled study | IG: 15  CG: 15 | IG: Mean age = 67.1±3.7 years; 10 females  CG: Mean age = 69.3±5.6 years; 7 females | IG: Online personalized exercise program in real time online via Skype under the supervision. The main part exercise program consisted of strengthening, balance, and stretching exercises. Online personalized exercise program.  CG: Waiting list | Each session was 40 to 45 min in duration for 3 days per week over 6 weeks, 18 sessions in total | Loneliness: Loneliness Scale for the Elderly-LSE | Before-and-after intervention | Synchronous tele-exercise seems to be an efective method to maintain their quality of life. It can make positive contributions to coping with loneliness and improve their mood. LSE scores worsened in the CG (p=0.016), there was no signifcant diference in the IG (p=0.162).There was a signifcant diference total scores of NHP (p<0.05). |
| Multicomponent interventions - VR and hands-on activities | The effects of a combination of 3D virtual reality and hands-on horticultural activities on mastery, achievement motives, self-esteem, isolation and depression: a quasi-experimental study | Fan et al. | 2022 | Aimed to explore the efects of a combination of three-dimensional virtual reality (VR) and hands-on horticultural activities on the psychological well-being. | Quasi-experimental study | IG: 32   CG: 30 | Mean age = 70.94 years (IG) and 69.83 years (CG) | IG: Combination of three-dimensional VR and hands-on horticultural activities. Participated in a gardening practice lesson in coordination with relative VR themes (Group intervention).  CG:  Scheduled activities, such as physical fitness, paper cutting, etc., without any gardening activities | 8 two-hour sessions were conducted once a week for 8 consecutive weeks | Perceived social isolation: Short-form scale of perceived isolation | Pre- and Post test | Signifcant post-intervention improvements in scores for self-esteem (β=2.18, P= .005) and mastery (β=1.23, P= .039), compared to the control group. All of 5 psychological well-being variables including  self-esteem (P<.001), depression (P=.004), isolation (P=.002), mastery (P=.002), and achievement motivation (P=.001) of experimental group had achieved signifcant improvement after intervention. |
| Application-based interventions | The first evaluation of a Mobile application to encourage social participation for community-dwelling older adults | Jansen-Kosterink et al. | 2020 | Assess the usability, end-user experience, and potential added value of this mobile application. | Observational study | 41 | Mean age = 73.4 years; 80% female | Social technology (mobile application) which encourages social participation. Stimulating older adults to visit local activities together with (new) friends. Six features: an inbox for messages, news, activities, information, tips, and friends. In the inbox, users received messages concerning the current news, activities, information, tips and friend requests but they could also send messages to their friends and to their personal coach. | Average used for 11 weeks (at least three months) | Loneliness: De Jong Gierveld loneliness scale (DJGLS) | Pre- and Post test | Level of experienced loneliness did not significantly change (p ≥ 0,442). 31% of the users showed a decrease in their level of experienced loneliness. Two-thirds of the users showed a clinically meaningful change on the SF-12 MCS or PCS. However, the increase scores on the MCS and the PCS of the SF-12 were not significant (p ≥ 0,062). |
| Community interventions - Group meetings | The Impact of a Naturally Occurring Retirement Community Supportive Services Program on Older Adult Participants' Social Networks: Semistructured Interview Study | Mills et al. | 2022 | Aimed to explore the experiences of 13 NORC-SSP residents who participated in Oasis Senior Supportive Living (Oasis) and how participating in NORC-SSP programming, specifically based on the Oasis model, influenced their social networks. | Qualitative descriptive approach | 13 | No age specified  92% (12) women | Oasis Senior Supportive Living (Oasis) is an innovative aging-in-place model that integrates health and supportive services for older adults in a NORC in partnership with older adults, building owners, researchers, and community partners. An on-site Oasis coordinator supports members to implement desired activities and services, including member-led activities such as craft circles, line dancing, potluck meals, or engaging community service providers to provide specific programming including exercise classes in communal meeting spaces | The Oasis program had been implemented approximately 1 year before the interviews. | Semistructured qualitative interviews | Single time point | Expand their social networks and improve their mental health and well-being. Ppportunity to build strong communities that provide deep, meaningful social connections that expand social networks. Participants reported the positive impact that Oasis had on their mental health and well-being, alleviating loneliness, and improving quality of life. |
| Video-conferencing interventions - Synchronous group interventions | The impact of a videoconferencing‐ımplemented program on older adults' psychosocial health in the COVID‐19 pandemic: an experimental study. | Yavuz & Şahin | 2023 | Explored the impact of a videoconferencing-implemented program on older adults’ psychosocial health during the COVID-19 pandemic. |  | IG: 40   CG: 52 | Aged 60 years and over  Mean age = 66.13 ± 5.13 years; 65.2% female | IG: Videoconferencing-ımplemented program. The program consisted of Sports, Skill Development, and Interactive Social Sciences courses, each of which was designed to last 45–60 min  CG: No Intervention | Three days a week for 8 weeks | Social Support: Multidimensional Scale of Perceived Social Support (MSPS)  Loneliness: Loneliness Scale for Elderly (LSE) - face-to-face interview technique | Pre- and Post test | Posttest score of the experiential group on the emotional loneliness subscale (LSE) was found to be significantly lower than that of the control group (p < 0.05). Could not conclude significant differences between the groups’ pretest and posttest LSE scores. Overall, the videoconferencing program was found to be efficient in providing older adults with psychosocial support. |
| Community interventions - Art and music interventions | The little intervention that could: creative aging implies healthy aging among Canadian seniors | Watson et al. | 2023 | Analyze the impact of an arts-based intervention targeting the elderly. | Randomized controlled study | IG: 130   CG: 122 | Mean age = 71 years;  83.7% female | IG: Arts-based intervention (mix of drawing, painting (acrylic, one-stroke, and water colour), collage, claywork, performance (creative movement and theatre), sculpting, and mixed media)  CG: No intervnetion | 2-h weekly sessions over a 16-month period | Loneliness: UCLA Loneliness Scale | Baseline and five times at the end of each block of artistic activities | Significant improvement in the well-being of those participating in the arts-based programme. This indicates less loneliness and depression, better mental health, more vitality, a higher level of emotional well-being, and improved quality of social interactions. |
| Videoconferencing interventions - Mixed | The Social and Cognitive Online Training (SCOT) project: A digital randomized controlled trial to promote socio-cognitive well-being in older adults | Funghi et al. | 2024 | Aimed to evaluate the efficacy of a multidimensional online intervention, as compared to non-specific cognitive stimulation, in improving socio-executive functioning in healthy older people. | Randomized controlled study | 60  IG: 30  CG: 30 | ≥64 years   Age = 71.8 years  IG: Age = 71.8 years; 18 female CG: Age = 71.7 years; 20 female | All interventions were delivered remotely via investigator-provided tablets.   IG:  Social and Cognitive Online Training - SCOT. Online social and executive intervention  consisting of two individual sessions of adaptive online cognitive training and one psychoeducational group session per week.  CG: A non-specific cognitive training group (CON). Non-specific and non-adaptive online cognitive intervention consisting  of two individual sessions of cognitive stimulation and one group session per week. | 8-weeks (24 sessions total, 60 min each) | Loneliness: Italian Social and Emotional Loneliness Scale (ISELS) | Baseline and at the end of the 8-week intervention | No significant changes were found in the ISELS scale. Found no significant effect on loneliness, which refers to the subjective feeling of being alone, separated or apart from others, regardless of the amount of social contact. |
| Web-based interventions - Individual interventions | Use and Effect of Embodied Conversational Agents for Improving Eating Behavior and Decreasing Loneliness Among Community-Dwelling Older Adults: Randomized Controlled Trial | Kramer et al. | 2022 | Investigate the effectiveness, the pathways to effects, and the mechanisms that underlay the use of an ECA targeting eating behavior and loneliness among older adults. | Randomized controlled study | 32 | 65 years or older  Mean age = 73.00 years (65-85); 18 (56%) female | IG: PACO service    CG: Waitlist group started PACO use after waiting for 4 weeks   PACO: Web-based eHealth service in which 2 ECAs (embodied conversational agent) engage in dialogue to provide motivation for improving eating behavior and decreasing loneliness | At intake, after 4 weeks, and after 8 weeks  IG: 8-week intervention immediately   CG:  4-week waiting list | Online questionnaires or optional interview   Loneliness: De Jong Gierveld loneliness scale | IG and CG: Baseline questionnaire (T0), after 4 (T1) and 8 (T2) weeks of use.  CG: Participants in cohort 2 were asked to complete an additional waitlist questionnaire (Tw) 4 weeks before T0. | PACO use did not lead to decreased loneliness (χ 2 2=0.02, P=.99). 4 participants mentioned making changes in their social network because of PACO. No significant differences over time in quality of life (χ22=2.99, P=.22). |
| Individual interventions - Trainer-administered | What is the ‘voltage drop’ when an effective health promoting intervention for older adults —Choose to Move (Phase 3)—Is implemented at broad scale? | McKay et al. | 2023 | To evaluate whether the CTM Phase 3 intervention increased older adults’ physical activity, mobility and health-related quality of life and reduced feelings of social isolation and loneliness. | Effectiveness-implementation pre-post study (observational study) | 1012 (Baseline) | Aged >60 years  Mean age = 72.9 years; 80.6% female | Choose to Move (CTM), an effective health-promoting intervention. CTM provides support for older adults to set personalized physical activity goals, and address barriers to physical activity. CTM participants receive social support from fellow participants and their activity coach. | During the first 3 months of the 6-month program, activity coaches provided participants: 1) one 60-minute one-on-one consultation, 2) five 60-minute motivational group meetings and 3) 3 check-ins (15 min, on average; once per month by phone, email or in person). During the last 3 months of the program, coaches provided less support (3 telephone calls). | Social isoaltion: three-item questionnaire adapted from two questions on social contact frequency  Loneliness: UCLA Loneliness Scale (UCLA-3) questionnaire | T0 (baseline), 3 (mid-intervention), 6 (end-intervention) and 18 (12- month follow-up) months | In all participants, social isolation and loneliness decreased during the intervention, but increased during follow-up. Social isolation score increased from baseline to 3 months (+0.8; 95% CI: 0.5, 1.1; indicating decreased feelings of social isolation) and remained higher at 6 months as compared with baseline (+0.5; 95% CI: 0.1, 0.8). Social isolation score decreased during the follow-up period such that values at 18 months were significantly lower than at 6 months and baseline (<75 years: -1.6; 95% CI: -2.0, -1.2;  >75 years: -2.5; 95% CI: -3.1, -2.0).  Loneliness score decreased from baseline to 3 months (-0.2; 95% CI: -0.4, -0.1; reduced feelings of loneliness) and remained lower at 6 months as compared with baseline (-0.3; 95% CI: -0.4, -0.1). Among older participants, loneliness score did not change significantly between baseline and 3 or 6 months. At the 18-month follow-up, younger participants’ loneliness scores were significantly higher than at the end of the intervention (+0.4; 95% CI: 0.2, 0.6). This resulted in no difference between loneliness scores at baseline and 18 months. Among older participants, loneliness scores also increased between 6- and 18-months (+0.6; 95% CI: 0.4, 0.9), which led to higher values at 18 months as compared with baseline (+0.4; 95% CI: 0.1, 0.7).   Health-related quality of life index score did not change significantly over time in either age group. |
